# Stem Cells on Biomaterials for Synthetic Grafts to Promote Vascular Healing

**DOI:** 10.3390/jcm3010039

**Published:** 2014-01-15

**Authors:** Patrick Babczyk, Clelia Conzendorf, Jens Klose, Margit Schulze, Kathrin Harre, Edda Tobiasch

**Affiliations:** 1 Department of Natural Science, Bonn-Rhein-Sieg University of Applied Science, Von-Liebig-Street 20, Rheinbach 53359, Germany; E-Mails: patrick.babczyk@h-brs.de (P.B.); margit.schulze@h-brs.de (M.S.); 2 Faculty of Mechanical Engineering/Process Engineering, University of Applied Science Dresden, Friedrich-List-Platz 1, Dresden 01069, Germany; E-Mails: clelia.conzendorf@htw-dresden.de (C.C.); jens.klose@zaft.htw-dresden.de (J.K.); harre@mw.htw-dresden.de (K.H.)

**Keywords:** stem cell, blood vessel, endothelial cell, smooth muscle cell, biomaterial, biopolymer, collagen, scaffold, surface modification, cross-linking

## Abstract

This review is divided into two interconnected parts, namely a biological and a chemical one. The focus of the first part is on the biological background for constructing tissue-engineered vascular grafts to promote vascular healing. Various cell types, such as embryonic, mesenchymal and induced pluripotent stem cells, progenitor cells and endothelial- and smooth muscle cells will be discussed with respect to their specific markers. The *in vitro* and *in vivo* models and their potential to treat vascular diseases are also introduced. The chemical part focuses on strategies using either artificial or natural polymers for scaffold fabrication, including decellularized cardiovascular tissue. An overview will be given on scaffold fabrication including conventional methods and nanotechnologies. Special attention is given to 3D network formation via different chemical and physical cross-linking methods. In particular, electron beam treatment is introduced as a method to combine 3D network formation and surface modification. The review includes recently published scientific data and patents which have been registered within the last decade.

## 1. Introduction

**Figure 1:**
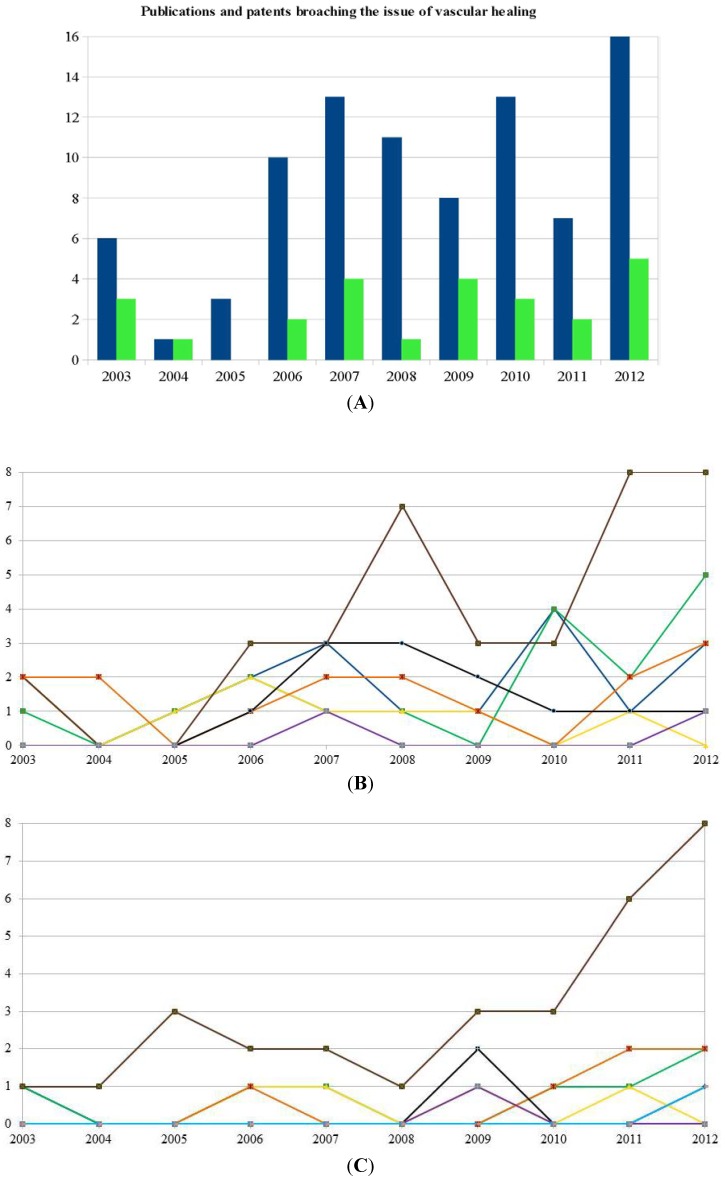
Publications and patents broaching the issue of vascular healing. (**A**) Publications (88) and patents (108) in the time period between 2003 and 2012 broaching the issue of vascular healing have been evaluated. The number of publications and patents is increasing after 2006 showing the growing interest in treating vascular diseases with stem cells and biomaterial. Blue: publications, green: patents; (**B**) Cell types for vascular healing approaches described in publications in the last decade. Embryonic stem cells were prominent in publications at the beginning of the decade. Their number decreased when in the iPS came up in the middle of the decade. Since iPS have a similar potency but cause less ethical problems and provide in addition the possibility for the future application of autologous cells, this was to be expected. Interestingly, the use of MSCs seems not to be affected by the development of induced pluripotent stem cell (iPS) cells. On the contrary, the use of this specific adult stem cell type is increasing. The reason for this might be the still unsolved risk of teratoma formation if endothelial cells (ECs) or IPS are used. ECs and smooth muscle cell (SMCs) have been described on a regular basis. The use of stem cells seems not to influence their utilization. Blue: EC, green: SMC, yellow: ESC, brown: MSC, orange: EPC, black: iPS, grey: other primary cells; (**C**) Cell types for vascular healing approaches described in patents during the last decade. The suggested used of the various cell types in patents follows the picture which can be seen in publications: The application of ECs is declining while the utilization of mesenchymal stem cell (MSC) which is the most prominent cell type. Blue: EC, green: SMC, yellow: ESC, brown: MSC, orange: EPC, black: iPS, grey: other primary cells.

This review summarizes original articles and patents from the last decade dealing with artificial replacements for damaged vessels with respect to tissue engineering methods and thus reflects the rising interest in this specific subject (Figure 1A). The focus is on the cell types and their characterization as well as on the used *in vitro* and *in vivo* 3D models. For a fundamental review on articles and patents dealing with the underlying cause of atherosclerosis and its treatment using bypass surgery or angioplasty with and without stents, we recommend the review of Limbach and colleagues [1].

Autologous arterial and venous grafts are commonly used and are the ideal source for small-diameter bypass grafts. However, if the patient does not have blood vessels of adequate quality, e.g., as a result of previous operations or collateral diseases, artificial grafts represent a promising alternative. The cells should be available in abundant supply, preferably from the patient himself to avoid undesired immune reactions. Thus, primary differentiated cells such as endothelial or smooth muscle cells are a reasonably good option if available. Guided tissue regeneration with undifferentiated or differentiated stem cells with the respective plasticity might be the alternative. Due to the limited availability of primary tissue cells, stem cells are of major interest for usage in grafts to promote vascular healing. This is mirrored in the publications and patents of the last decade (Figure 1B,C).

Stem cells can be divided into three major groups: pluripotent embryonic stem cell (ESCs), induced pluripotent stem cells (iPS) and adult stem cells (ASCs) of various plasticities. All groups share the ability of self-renewal, a major advantage compared to primary tissue cells, but they differ in their capacity to differentiate into the various tissue lineages. While ESCs and iPS are pluripotent, meaning they can differentiate into any cell type of the three germinal layers, ASCs are only multipotent, thus have a limited ability to differentiate towards several lineages only [2,3].

Embryonic stem cells were the prominent cell source in publications at the beginning of the decade but the numbers decreased when Takanashi and Yamanka described a method to induce pluripotency in somatic cells, creating the so called iPS [4,5]. Since iPS have a similar potency but cause fewer ethical problems and provide in addition the possibility for the future application of autologous cells, there is a rising interest in this specific cell type (Figure 1B,C). Interestingly the use of mesenchymal stem cells (MSCs) was not affected by the hype on iPS cells for future applications in regenerative medicine. MSCs, which belong to the group of adult stem cells, are multipotent and represent an interesting source for the use on vascular grafts since they can be differentiated in the desired cell types. Progenitor cells, such as endothelial progenitor cells (EPC) are unipotent, meaning they can only differentiate into one specific cell type, e.g., endothelial cell (EC). EPCs can be obtained from peripheral blood (PB-EPC), bone marrow (BM-EPC), umbilical cords (UC-EPC) and umbilical cord blood (UB-EPC) and named accordingly. According to the composition of blood vessels, primary endothelial cells and smooth muscle cells can be used as well in grafts to promote vascular healing. The isolation of ECs and SMCs is always accompanied with the destruction of the tissue (e.g., arteries and veins) and therefore limited in humans. In addition, the collectable amount of cells is low; thus, it is time consuming to get an appropriate amount of cells for vascular grafts. This might be the reason why the last mentioned cells types, although they have been used for quite some time, have never peaked but are still used on a regular basis (Figure 1B,C).

Even though the best cell type for vascular grafts and other clinical applications is still unclear, more than 3000 clinical trials have been applied so far utilizing stem cells to treat various diseases [6]. Most of these treatments are still in phase I or phase II, and most of them focus on cancer (>2500), and not tissue engineering but nevertheless they provide some new insight and improvement for regenerative approaches. However, stem cells seem to have only short-term positive effects within their tissues and data on long-term benefits are missing for nearly all stem cell therapies. The stem cells seem to have only paracrine effects on the neighboring progenitors and somatic cells, and are lost within weeks of the application, presumably via apoptosis [7]. This is the polar opposite of what was expected, namely that stem cells will repair the damaged tissue or organ by differentiating into the cells which form and be functional in these tissues and organs. However, how to stimulate and induce stem cells to achieve the desired differentiation and/or repair the tissue seemingly cannot currently be pinned down. Therefore, addressing key molecules which regulate and determine stem cell fate is vital to improve above mentioned poor knowledge on what happens in tissue regeneration approaches with these cells and thus will be summarized and discussed in this review.

## 2. Stem Cells for Vascular Grafts

The focus of the chapter is the characterization of various stem cell types differentiated towards endothelial and/or smooth muscle cells. For the characterization of the undifferentiated stem cells with their specific markers, the review of Calloni and coworkers is recommended [8].

### 2.1. Embryonic Stem Cells

Embryonic stem cells are derived from the inner cell mass of blastocysts, which subsequently forms the embryo. As already mentioned, the pluripotency of these cells is their major advantage. However, this advantage is coupled with several disadvantages, such as ethical problems, due to the cell source and teratoma formation due to the fact that all cells will never undergo the induced differentiation process. In the past, most ESCs and embryonic germ cells were derived from human and mouse embryos. They have been characterized thoroughly defining a well defined set of specific markers. For a review on the marker of undifferentiated ESCs or the developmentally distinct and different markers of embryonic germ cells, the publications of Calloni [8] and Shamblott [9] are recommended. Recently, ESCs from mouse, human and rabbit have been used to construct tissue-engineered blood vessels (TEBV) or tissue-engineered vascular grafts (TEVG). To do so, the ESCs have been differentiated towards endothelial cells or smooth muscle cells by several groups.

#### 2.1.1. Endothelial Cell and Smooth Muscle Cell Markers Expressed in Differentiated ESCs

To obtain cells for synthetic grafts promoting vascular healing, the ESCs have to be differentiated towards the desired lineages, namely ECs and SMCs. Subsequently, the cells have to be analyzed due to their expression pattern of EC and SMC marker. Depending on the strategy used, which is defined by differentiation media and the culture model, the groups achieved different results. Three representative strategies and their results are introduced here [10,11,12]. The three groups agreed on the EC marker PECAM-1 and the smooth muscle marker aSMA as key marker. Nakagami and colleagues showed that differentiated ESCs seeded on matrigel additionally secreted the growth factors VEGF, bFGF, HGF, TGF-β and Ang-1 [11]. The group of Ferreira cultured hESCs in EGM-2 medium with either the addition of VEGF-A_165_ or PDGF-BB and showed that these differentiated cells either expressed the EC markers PECAM-1, KDR/Flk-1, VE-cadherin, vWF or the SMC markers aSMA, SM myosin HC and calponin, respectively [12]. The influence of pulsatile flow with additional VEGF investigated by Huang resulted in an EC phenotype, while the lack of VEGF under the same conditions resulted in SMC phenotype [10]. In summary, the groups agree on VEGF as the major factor during both lineage commitments independent on the cell culture system while using ESCs as cell source.

#### 2.1.2. Embryonic Stem Cells for *in Vitro* 3D Culture Models

To transfer a two-dimensional model into a three-dimensional model, two major strategies are pursed. In order to grow differentiated cells in gelatinous protein mixture, matrigel is the most commonly used material. It is used to investigate sprouting as a first step towards the formation of capillaries or the application of shear stress during flow to improve endothelial differentiation. An example of the former is the publication of Nakagami, whereas Huang used differentiation from murine embryonic stem (ES) cells to vascular wall cells by mechanical stress loading in microporous tubes made of segmented polyurethane [10,11]. The oriented cells exhibited endothelial-like appearance and showed orientation into the direction of flow. Abilez and colleagues used a combination of both techniques and investigated the influence of pulsatile flow on undifferentiated mESC in a three-dimensional matrigel [13]. They were able to show that the cells constrained to the wells of the culture system, moved in unison with the applied flows, and were not washed downstream due to the additional application of the matrigel. Obviously this has to be repeated with the differentiated cells for further use.

#### 2.1.3. Embryonic Stem Cells for Vascular Grafts in Animal Models

The major animal model to investigate the potential of ESCs for vascular healing is the mouse. In this model, xenotransplantation using ESCs of other origin is also common. Large animal models such as pigs, dogs, and sheep are used as well. The focus of these experiments is cell recruitment and the ESCs’ fate in these animals. Next to the above-mentioned molecular studies on the expression patterns of specific EC and SMC markers, these investigations include mainly histological and immunohistochemical experiments. Ferreira and colleagues confirmed the vascular potential of hESCs by implanting these cells grown on matrigel into mice, which resulted in the formation of a microvasculature [12]. Xiong and co-workers cultured hESCs-derived ECs and SMCs in a 3D fibrin gel and transplanted the graft into a myocardium infarction site of pigs and observed ventricular remodeling [14]. Shen and colleagues differentiated rabbit ESCs towards SMCs [15]. They transplanted a seeded PGA-scaffold into mice and showed the lining of endothelial cells on the lumimal surface and the presence of SMC and collagen in the wall.

#### 2.1.4. Patents on Embryonic Stem Cells for Vascular Grafts

Interestingly, the current major focus of ESCs in patents is not the use of these cells in tissue engineering but the production of an extracellular matrix to promote wound healing and thus inhibit scarring. A typical example is the patent of McDevitt who developed a decellularized scaffold from embryonic stem cells to promote vascular healing [16]. Similarly, Yeryemyeyev produced a cardiomyocyte-matrix with the help of hESCs grown in defined medium to produce an extracellular matrix [17]. Nevertheless, tissue engineering for vascular organogenesis has also been patented by Hayashi for cultured ESCs or embryonic stem cell-derived embryoid bodies on growth factor-reduced matrigel [18]. 

### 2.2. Induced Pluripotent Stem Cells

In 2006, Takahashi and Yamanaka induced pluripotency in mouse embryonic and adult fibroblasts via viral introduction of the transcription factors Oct3/4, Sox2, c-Myc and Klf-4 [4,5]. They named the resulting cells “induced pluripotent stem cells”, iPS. The obtained cells showed ESC morphology and expressed typical ESC markers. When injected into blastocytes the iPS were incorporated into the development of the embryo. Injection into mice subcutaneous resulted in iPS derived tumors which contained cells from all three germ layers. Wernig and colleagues could show that iPS are similar but not identical to ESC regarding methylation and chromatin state [19]. The iPS were able to form live late-term murine embryos. Yu and colleagues induced pluripotency and an ESC phenotype in human somatic fibroblasts characterized via screening of specific genes and surfaces markers and differentiation into all germ layers [20]. With application of nonintegrating adenoviruses transiently expressing the stem cell factors Oct3/4, Sox2, Klf4, and c-Myc the induction of pluripotency via potentially carcinogenic retroviruses was overcome [21]. However, the potential for tumor formation also originates from the pluripotency inducing four transcription factors. Several approaches have been conducted to reduce the number of necessary transcription factors. Kim and co-workers saw that murine ASCs expressed higher endogenous levels of Sox2 and c-Myc and therefore conducted the pluripotency induction only using the factors Oct3/4, and c-Myc or Klf4 [22,23]. The patented invention of Yamanaka disclosed similar methods inducing pluripotency in mammalian cells using the factors Oct3/4 or Nanog, introduced by viral vectors or nucleic acids encoding the latter two factors [24]. In the meantime, iPS induced with two factors (2*F*; Oct3/4 and Sox2) and even with only 1*F* (Oct3/4) have been produced where the amount of necessary factors is dependent on the cells source, progenitor cells or even stem cells, which need fewer transcription factors for the reprogramming process.

#### Induced Pluripotent Stem Cells for Vascular Grafts

Choi and colleagues reprogrammed human iPS cell lines from fibroblasts of different origin with Oct3/4, Sox2, Nanog and Lin26 [25]. In a co-culture system with murine bone marrow stromal cells they characterized the iPS for their differentiation potential and showed that the cells can differentiate into CD34/CD43 positive hematopoietic progenitor cells and ECs (PECAM-1 positive and CD43 negative). The treatment of limb ischemia was investigated by Lian and colleagues using comparison studies of the survival rate and differentiation potential of BM-MSC and human iPS-cell-derived MSCs [26]. The MSCs and iPS-derived cells were transplanted into mice and both cell types attenuated the ischemia. Both cell types promoted vascular and smooth muscle regeneration, with the positive effects of iPS-derived cells being the stronger one. Hibino and colleagues differentiated mouse iPS and produced a cell sheet as tissue-engineered vascular graft using temperature-responsive dishes [27]. They seeded the cells onto a biodegradable scaffold composed of polyglycolic acid-poly-l-lactide and poly(l-lactide-*co*-ε-caprolactone) and implanted it as interposition grafts into the inferior vena cava of mice. The cells expressed EC markers and histological investigations showed endothelialization and an inner SMC layer. The seeded iPS cells exerted a paracrine effect to induce neotissue formation in the acute phase and were lost over time.

### 2.3. Mesenchymal Stem Cells

Mesenchymal stem cells belong to the group of adult stem cells which are multipotent. They represent an increasingly investigated cell source for the use on vascular grafts (Figure 1B). These cells can be isolated from several tissues including wisdom teeth, peripheral blood, neonatal tissues (specific parts of the placenta or the umbilical cord), bone marrow and fat, the latter two representing the most common sources [28,29,30,31,32,33,34]. There is a confusing amount of so-called specific markers to define this stem cell type, which, in addition, varies to some extent depending on their tissue origin. Thus, most researchers agree and rely in the meantime on the minimal criteria as defined by the International Society of Cellular Therapy. To do so, MSCs should adhere to plastic under standard culture conditions, they must be able to differentiate into osteoblasts, adipocytes and chondroblasts under standard *in vitro* differentiating conditions, as confirmed by specific stainings. Additionally, a positive expression pattern (>95% of the cells) of CD73, CD90, CD105 is required as well as the absent expression (>98% of the cells) of CD45, CD34, CD14 or CD11b, CD79 or CD19 and HLA-DR [35]. Recent publications showed that, next to the cell source, the MSC population itself seems to be a heterogeneous mix of pre-committed and differentially methylated cells. Purinergic receptors and PTHrP are marker to define and distinguish these cells within a population [36,37,38]. The amount of the intensively investigated bone marrow-derived MSCs is rather limited which led to a shift of interest in an alternative source where higher amounts can be obtained, harboring a higher yield of cells in recent years. Adipose tissue-derived stem cells (ATSCs) are therefore of major interest. Here, the MSCs can be collected from the waste product of liposuction surgery applied on healthy donors [39,40]. Undifferentiated MSCs and MSCs differentiated towards ECs and SMCs are used for experiments in vascular research. They are investigated as stabilizing supporter cells for vascular networks or directly in TEVG. Cell culture models as test systems for preliminary biocompatibility studies and *in vivo* models, both with and without differentiation studies, are the main approaches to investigate the potential of MSCs to form vascular structures. 

#### 2.3.1. Endothelial Cell and Smooth Muscle Cell Markers Expressed in Differentiated MSCs

The differentiation and angiogenic potential of hMSC is evaluated under two diametrically opposed aspects. Some researchers are interested in understanding angiogenesis to be able to inhibit tumor microvessels development as cancer treatment. Others are interested in understanding angiogenesis for future tissue replacement strategies in regenerative medicine. Understanding the underlying basic mechanism is the common ground for both. Thus, selected publications of the former are mentioned within this paragraph as well. One example for this is the work of Birnbaum and colleagues who differentiated hMSCs under the influence of glioblastoma-conditioned medium and confirmed the results of EC and SMC markers expression via tube-formation assays [41]. The tumor-conditioned hMSC expressed CD151, VE-cadherin, desmin, alpha-smooth muscle actin, nestin and nerval/glial antigen 2 (NG2). Since no expression of von-Willebrand factor (vWF) and smooth myosin could be detected, these findings indicate a GBM-dependent differentiation of hMSC into pericyte-like cells, rather than into endothelial or smooth muscle cells. Kim detected EC and SMC markers (CD34, CD31, KDR, VEGF and alpha-smooth muscle actin) with immobilized bFGF using a decellularized natural matrix [42]. Duffy and colleagues used a collagen based matrix to establish a nascent vascular network *in vitro* within this glycosaminoglycan matrix [43]. To do so this group seeded the scaffold with MSCs and showed that the cells expressed PECAM-1 and VCAM but there was no increase in Tie-2 and vWF expression and the alpha-smooth muscle actin expression fell.

#### 2.3.2. MSCs for *in Vitro* Differentiation Assays

The differentiation of MSCs can be influenced by mechanical and physical stimulation such as elasticity and geometry, shear stress and pressure, or pH and oxygen, and various soluble and bound chemical factors from the matrix. For a review on this, see Schulze and Tobiasch [6]. Exemplary EC and SMC differentiation assays with the center of attention on mechanical and physical stimulation are listed in the following. Song and colleagues focused on mechanical properties such as flexibility and an elastic porous tubular structure of poly(1,3-trimethylene carbonate), PTMC, cross-linked by gamma-irradiation in an inert nitrogen atmosphere with an interconnected pore network prepared by particulate leaching for tissue engineering of small-diameter blood vessels which they tested for biocompatibility [44]. Tsigkou achieved anastomosis and human-derived vessels with long term stability where the majority of the graft vasculature had been functionally remodeled with host cells while generating vascularized bone grafts using mesenchymal stem cells from human bone marrow and umbilical cord-derived endothelial cells in the very stiff material derived from decellularized allogeneic bone grafts [45]. Gong on the other hand developed a two-step model to engineer small-diameter vessels by seeding BM-MSCs onto a very soft fibronectin and collagen matrix showing differentiation into SMC under the influence of soluble factors (TGF-β1, PDGF-bb) and cyclic strain. Histological and molecular examinations showed similarities to native vessels [46].

#### 2.3.3. MSCs for Vascular Grafts in Animal Models

For *in vivo* models investigating the potential of MSCs in vascular healing, TEVGs have been developed by using natural, natural-derived and artificial materials for scaffolds, with collagen and matrigel being the most common materials. A typical example for the former is the work of Kim and coworkers who successfully cultured hATSCs and various other cells on a decellularized human extracellular matrix from adipose tissue investigating the usefulness of such a natural scaffold material [47]. Zhao and colleagues developed a tissue-engineered artery using autologous bone marrow derived mesenchymal stem cells (MSCs) differentiated into (SMCs)-like cells and endothelial cell (ECs)-like cells, seeded onto decellularized ovine carotid arteries and interposed into the carotid arteries in an ovine host model [48]. The group of Dufourcq modified murine MSC genetically to secrete frizzled-related protein-1, a modulator of the Wnt/Fz pathway and showed that they recruited ECs and SMCs to enhance neovessels maturation in a mouse engraftment model [49]. Altman, using a natural-derived artificial scaffold, seeded hATSCs on a silk fibroin-chitosan (SFCS) scaffold in a murine soft tissue injury model and showed positive expression patterns of fibroblasts (hsp47), EC (vWF) and SMC (alpha-smooth muscle actin) [50]. Chung and his group observed network formation in a three-dimensional matrigel by tracing ATSCs from rats via nanoscaled gold spheres [51]. Matsumura and colleagues seeded tissue-engineered vascular autografts with bone marrow cell for future repair of complex cardiac defects and successfully tested the graft transplanted in the inferior vena cava of dogs for various early and late EC and SMC marker [52]. Cho and Lim used a hybrid biodegradable polymer scaffold from poly(lactide-*co*-epsilon-caprolactone) (PLCL) copolymer reinforced with poly(glycolic acid) (PGA) fibers to produce a tissue-engineered vascular patch by using autologous bone marrow-derived cells in the dogs [53,54]. While Mendelson and colleagues developed a two-step model using ovine blood-derived endothelial progenitor cells (EPCs) and bone marrow-derived MSCs seeded onto poly-4-hydroxybutyrate (P4HB)-coated polyglycolic acid (PGA) nonwoven biodegradable mesh scaffolds first applied to flow in a bioreactor and later *in vivo*, using cell-seeded patches implanted in the sheep pulmonary artery [55]*.* A very different question was addressed by Derval and coworkers who used murine MSCs and EPCs on coated muscle patches as therapy approach for distance of myocardium infarction (MI) in a mouse model. They showed that only MSCs exhibited the capacity to invade scar tissue [56].

### 2.4. Endothelial and Smooth Muscle Progenitor Cells

The plasticity of stem cells always encloses the risk of tumor formation which to some extent is linear to the potency of the cells. In addition this flexibility to differentiate in several lineages also contains the risk of undesired spontaneous differentiations towards the wrong tissue type. On the other hand, the use of fully differentiated tissue cells is limited due to the low cell amount achievable and problem with long-term culture. This makes precursor cells which still have some although limited differentiation potential combined with a reasonable good proliferation capacity an interesting cell type.

When the group of Ashara and later Shi discovered the existence of endothelial progenitor cells (EPCs) derived from bone marrow (BM-EPC), a new cell type was available which seemed to be an ideal cell source for vascular healing approaches providing an appropriate amount of ECs and SMCs [57,58]. EPCs can also be obtained from peripheral blood (PB-EPC), umbilical cord (UC-EPC), and umbilical cord blood (UB-EPC). They can be differentiated into ECs in the presence of growth factors such as VEGF, basic fibroblast growth factor (bFGF), and insulin-like growth factor-1 (IGF-1) [59,60]. Interestingly, EPCs circulating in peripheral blood (PB-EPC) settle on denuded endothelial sites within the vessels. Shirota and coworker mimicked this natural behavior of the cells [61]. They isolated EPCs from human blood and performed *in vitro* studies with the cells and a microporous segmented polyurethane film coated with a photo-reactive gelatin layer and applied to flow shear stress. They observed EPCs fully covering the graft elongating and aligning themselves with the direction of the flow similar to native EC.

#### 2.4.1. EC and SMC Expressed in Differentiation Assays of Vascular Precursor Cells

The definition of specific marker in vascular precursor cells is interesting because they are a defined natural stage between the non- or little committed stem cells and the fully differentiated tissue cells. Since the appearance and disappearance of so-called stage specific markers is always difficult due to the process being a fluent event and not a sudden onset after the initiation of differentiation towards the desired lineage, a defined set of minimal criteria as for MSC would be desirable but has not been agreed upon until now.

Le Ricousse-Roussanne and Amini isolated progenitor cells and detected EC and SMC markers, while Davis injected peptides that recruited progenitor cells expressing EC and SMC markers [62,63,64]. The group of Le Ricousse-Roussanne obtained cells from cord blood and investigated if these progenitor cells are a source for vascular therapy. They could show that cobblestone-like cells expressed endothelial cell markers, while spindle-shaped cells expressed SMC markers. Amini compared EPC from rabbit peripheral blood (PB-EPC) and bone marrow EPC (BM-EPC). PB-EPC displayed EC markers like CD31 and a high angiogenic potential in a 3D matrix. Co-cultured with MSCs, the cells expressed high levels of osteogenic and vascular markers (AP, bone morphogenic protein 2, VEGF). BM-EPC did not express PECAM-1, but it did express smooth muscle markers [63]. Davis injected self-assembling peptides in the myocardium and detected recruitment of progenitor cells expressing EC and SMC markers in the microenvironment of the nanofibers produced by the peptides [64].

Differentiation studies were conducted by Sainz, Sreerekha and Wu [65,66,67]. They also showed expression of specific EC- (PECAM-1, VE-cadherin, vWF) and SMC (aSMA, calponin, SMA, HC) markers in the resulting cells. Allen and co-workers engineered a functional endothelium on a polymer by using EPCs from porcine peripheral blood [68]. The EPCs differentiated into ECs and expressed EC markers (vWF, PECAM-1, VE-cadherin) as well as ac-LDL uptake. In a co-culture model, the influence on porcine aortic smooth muscle cells (PASMC) was investigated and showed a decrease in PASMC proliferation. Cherqui used ECs and EPCs and transduced them using viral macrophage inflammatory protein-II (vMIP-II), which is immunosuppressive and proangiogenic [69]. They engineered vMIP-II lentiviral gene vectors and transplanted matrigel templates and observed functional and branching vessels with associated smooth muscles. Schmeckpeper cultured BM-EPC-derived ECs on collagen and BM-derived SMC on fibronectin in order to investigate vector ability to track vascular differentiation, showing EC markers VE-cadherin and expression of eNOS and SMC markers aSMA, calponin and SM myosin HC [70]. Fioretta and colleagues investigated the influence of substrate stiffness on progenitor cells fate [71]. They seeded EPCs from peripheral blood on soft, intermediate, and stiff fibronectin-coated polyacrylamide gels and analyzed adhesion (vinculin staining), proliferation (BrdU), phenotype (CD31, aSMA, FACS analysis), and collagen production. They observed that adhesion and proliferation increased with substrate stiffness but no influence on change of phenotype could be seen. Clever and colleagues did an inhibition study using EPCs and hCASMCs [72]. They showed that application of paclitaxel, sirolimus and iopromide inhibited proliferation and migration in a dose-dependent manner.

#### 2.4.2. Endothelial and Smooth Muscle Progenitor Cells for Vascular Grafts in Animal Models

Basic studies and the potential of vascular progenitor cells to promote vascular healing has been investigated in small and large animal models with EPCs being the major focus of attention, but co-recruitment of EPCs and SMCs progenitor cells has been examined, too. Fioretta and colleagues investigated the fate of EPCs on scaffolds with different degrees of stiffness [71]. Cottone registered a patent using a drug delivery graft recruiting EPCs for restoring an endothelium [73]. Melero-Martin co-cultured EPCs from various sources (human umbilical cord blood, peripheral blood, and human saphenous vein smooth muscle cells (HSVSMCs)) on matrigel and implanted the grafts into mice to form vascular networks [74]. Griese and colleagues isolated EPCs from blood to re-endothelialize balloon-injured carotid arteries in rabbits with and without bioprosthetic grafts [75]. They observed inhibition of neointima deposition. Similarly, he and colleagues transplanted autologous circulating endothelial progenitor cells prelined on the luminal surface of *in situ*-formed collagen type I meshes as extracellular matrix wrapped with a segmented polyurethane thin film in canine arteries [76]. The graft should serve as small-diameter-vessel prostheses and ensure nonthrombogenicity. Allen used EPCs from circulating blood to engineer a functional endothelium on poly(1,8-octanediol-*co*-citrate), a hemocompatible and biodegradable elastomer for vascular tissue engineering in a pig model [68]. Yu and coworker showed the simultaneous recruiting of ECs and SMCs progenitor cells for *in situ* blood vessel regeneration in rats [77].

### 2.5. Patents on Adult Stem and Precursor Cell Isolation and Maintenance

This paragraph summarizes in brief patents on isolation, characterization, proliferation, migration and differentiation of adult stem and precursor cells. Some multipotent stem cells next to MSC are discussed because the application of various precursor and adult stem cells, mainly MSCs increased in patents during the last ten years (Figure 1C). Some differentiations of applications diverse form the area of this review will be mentioned briefly because they provide insides into the more general aspects of the above-mentioned features and thus will be beneficial for researcher interested in the topic of this review. Scaffolds will be introduced here only in a few words because they are discussed thoroughly in the second part of this review.

The patented isolation procedures can be divided into those which describe specific mechanical techniques and those which rely on chemical procedures to improve the outcome. Noishiki patented a mechanical method to harvest stem cells of bone marrow from long tubular bones [78]. Muschler suggested another method to do the same [79]. Sciorra’s invention relates to a culture system suitable for high throughput screening applications using a 3-dimensional tissue models that may be used to identify new drugs [80]. Harman’s invention is to prepare a stem cell composition from a collagen-based tissue, such as adipose tissue, isolated from a patient and subsequently provide it to a site of injury [81]. Kieda described a method to isolated human precursors of endothelial cells to create cell lines for future application [82]. Ma suggested seeding MSC on a planar surface or a porous 3-D scaffold comprised of non-degradable polymer such as poly(ethylene terephthalate) (PET) and grow the cells under physiological or low O_2_ tension to maintain their self renewal capacity [83]. Xianqun has patented a hermetic material, which is a soft silica gel or a sticky plastic film [84]. The material is supposed to be put under negative pressure so that the stem cell suspension completely reaches the inside of the porous biomaterial for fusion. To isolate or improve the proliferation of adult stem cells, several inventors followed strategies using peptides and cytokines. Gostjeva’s invention provides methods identifying molecules to modulate proliferation and/or migration of metakaryotic stem cells disorders, such as blood vessel wound healing disorders, including restenosis [85]. Amoh claimed nestin expression as a marker for endothelial cell proliferation [86]. Lee’s invention provides a method for inducing *in vivo* migration of stem cell using a biodegradable scaffold with various chemotactic factors [87]. Hantash has differentiated MSCs towards fibroblasts with various cytokines on various scaffolds, namely hydrogel, PLGA, collagen gel, matrigel, spongastan, and fibronectin [88]. Guoqi has patented the use of facial masticatory muscles genetically altered towards cardiac muscles cells, which have the same origin for the progenitor cells, on a cell membrane technique [89]. Nanxue’s invention relates to a method used to process directional-inducing ATSC towards adipose and endothelial cell differentiation and the combination of the cells with a biomaterial to remedy soft tissue defects [90]. Shortkroff has patented a double-structured tissue implant composed of a soluble collagen in combination with a non-ionic surfactant positioned within the primary scaffold for differentiation and growth of mesenchymal and bone marrow stem cells [91]. Park attempted to achieve growth and differentiation of stem cells by modifying the surface of the polymeric scaffold using the decellularized extracellular matrix directly derived from specific tissue cells [92]. Aicher suggested the same for mesenchymal progenitor cells of the umbilical cord [93]. Hamada introduced a carbonate apatite-collagen sponge comprising the SVVYGLR peptide which can stimulate the proliferation of mesenchymal and dental pulp cells. This is supposed to be useful a biomaterial for the regeneration of bone marrow or dental pulp [94]. Cho found that scaffolds with a high content of sulfated glycosaminoglycans facilitate cell adhesion and provide biomimetic surface environments that are effective for growing and differentiating stem cells [95]. Komeno used anaplastic mesenchymal stem cells embedded in a gel forming material such as collagen and hyaluronic acid [96]. Similar, Huang claimed that stem cells display improved viability when grown in a cell tissue gel that contains collagen and hyaluronan at particular weight ratios [97]. A modification of this is the patent of Abatangelo who suggested using differentiated bone marrow stem cells seeded on an extracellular matrix and a second component biodegradable matrix consisting of a hyaluronic acid ester [98]. Choi described a medical composite biomaterial comprising collagen and a hyaluronic acid derivative using stem cells derived from a mammal umbilical cord [99]. This is very similar to the suggestion of Komeno who patented anaplastic mesenchymal stem cells embedded in a gel forming material such as collagen and hyaluronic acid [96]. A two component scaffold was also favored by Min who patented the use of an alginate coated fibrin/ha composite scaffold [100]. Another two component scaffold was patented by Shimizu [101]. He wants to use an artificial blood vessel comprising a tube made of a supporting skeletal material and a collagen layer of ultrafine fibers at the outside of the tube. Some patent holders suggest not using the stem cells itself but rather a secreted product thereof. The strategy of using conditioned medium of MSCs for multiorgan failure was tracked by Westenfelder [102]. Arai suggested a new method for producing type 14, 12 collagen for wound healing from genetically altered stem cells [103].

## 3. Primary Tissue Cells for Vascular Grafts

Some researchers prefer primary vascular cells instead of precursor or stem cells. This might be due to the fact that these cells do not feature the tumourigenic potential typical for cells with a higher plasticity. On the other hand this advantage is abolished by the restricted access to the cell sources in humans, namely umbilical cords, aorta, coronary arteries and veins, and the fact that the differentiated primary cells also lack the self renewal capacity of the stem cells. Thus the use of these cell types in constant but rather low over the last decade (Figure 1B,C).

### 3.1. Endothelial Cells

Endothelial cells constitute the inner wall of blood vessels by forming a monolayer. They form a barrier for erythrocytes but provide the transition of hormones, oxygen, nutritional factors, and other proteins and take part in the regulation of blood pressure through the enzyme endothelial nitric monoxide synthase (eNOS) and the resulting production of nitric monoxide (NO) causing vasodilatation of the vessels. Heme oxygenase 1 is another important enzyme producing carbon monoxide and modulating EC apoptosis [104,105,106]. An additional feature is the production of clotting factors to influence the viscosity of the blood. Further ECs take part in immune response by their ability to bind leukocytes and activate them. The monolayer formed by ECs is often described as “cobblestone pattern” due to their polygonal shape. ECs express specific proteins, which are used to identify them via histological and immunohistochemical stainings as well as expression patterns on the RNA and protein level, including platelet derived adhesion molecule-1 (PECAM-1/CD31), von Willebrand factor (vWF), vascular endothelial cadherin (VE-cadherin), and the VEGF-receptor-2 (kinase-insert domain receptor (KDR)/fetal liver kinase-1 (Flk-1)). Sources of ECs are capillaries, arteries, aortic vessels and veins of a multiplicity of organisms. They can be isolated via digestion of tissue or by scraping the monolayer of vessel. Jaffe and colleagues described a procedure to isolate and expand ECs from human umbilical cords (therefore named human umbilical cord endothelial cells, HUVEC), whereas Voyta isolated primary ECs from bovine capillaries and aortic vessels via cell sorting [107,108]. Knighton patented a method using sponges in wounds to isolate microvascular ECs. The cells surrounding the wound and can invade the sponge, which can be degraded subsequently [109].

### 3.2. Smooth Muscle Cells

Smooth muscle cells of the vasculature are located in the media of arteries and veins. The principal task of SMC in arteries is to regulate the blood pressure by vasodilatation and vasoconstriction and to provide mechanical strength of the vessels [110]. SMCs are spindle-shaped and also express specific proteins like alpha-smooth muscle actin (αSMA), smooth muscle myosin heavy chains, and calponin. Those proteins can also be used to identify SMCs via histological and imunnohistochemical staining as well as expression patterns on the RNA and protein level. The maintenance of homogeneous SMC populations is more extensive than EC populations. Due to their location in the media of vessels surrounded by elastic fibers like elastin, digestion of the tissue or the outgrowth of vessel rings and tissue pieces must be performed. The challenge is to maintain vessels without an inner monolayer of ECs, which can be done by digestion, e.g., collagenase, conducted by Jaffe for isolation of HUVECs [107]. Additionally, the adventitia has to be removed to ensure an outgrowth of the SMCs, which can last a long time. Another threat to homogeneous populations of SMCs is the cross-border population with fibroblasts, which are similar in morphological shape. SMC isolation has been conducted from various animal models and different human tissues (e.g., human coronary artery, human umbilical artery/vein). Ray and co-workers isolated SMCs from murine aorta, whereas Ribeiro isolated SMCs from human umbilical cords [111,112].

### 3.3. Endothelial Cells and Smooth Muscle Cells for Vascular Grafts

ECs strictly grow as monolayers *in vivo* and therefore it is required to transport the cells from a two-dimensional culturing system into a three-dimensional environment like a graft. Several studies have been concerned with the investigation of co-culture models on a three-dimensional matrix and the influence of the matrix. This research is often connected to animal models in order to observe the potential of vascular healing. Co-culture of ECs and SMCs on grafts mimic *in vivo* conditions and enhance vascular properties. Fillinger and colleagues co-cultured ECs and SMCs on opposite site of a porous membrane and observed the formation of gap junctions between each other [113]. The effect of direct contact was investigated by Wallace and colleagues, co-culturing ECs and SMCs in comparison to a co-culture on porous membranes [114]. Direct contact led to low levels of intercellular cell adhesion molecule-1, vascular cell adhesion molecule-1 and E-selectin surface protein, all necessary for monocyte transition through EC layer during atherosclerotic inflammation. Their conclusion is that direct contact of ECs and SMCs in co-cultures on grafts render graft ECs more resistant to inflammatory signals. Chen and colleagues investigated the effect of copolymers on the proliferation of ECs and SMCs [115]. They showed that vascular cells grew well on moderately hydrophilic membranes. The working group of Kim BS seeded HUVEC on decellularized human adipose-tissue-derived extracellular matrix sheets and observed spreading of the cells and integration into the matrix [47]. Baguneid went one step further and developed a tissue-engineered blood vessel on a collagen scaffold [116]. ECs and SMCs were seeded on a collagen matrix and transplanted into pigs. The TEBV showed endothelialization and neo-vascularization. Boni replaced left pulmonary arteries of lambs with porcine small intestine sub-mucosal biomaterial and confirmed the presence of SMCs in the medial layer [117]. Callegari implanted porous collagen scaffolds on cryo-injured rat hearts [118]. The collagen scaffolds were absorbed and became populated by new arterioles and capillaries. The cells expressed EC and SMC markers and no cardiomyocyte markers. Similarly, Saif developed biodegradable PGA scaffolds that released VEGF, HGF, and angiopoietin-1 as combination or sole [119]. In a murine hindlimb ischemia model they found out that increasing migration of vascular progenitor cells took place when all factors were secreted. The scaffold was vascularized with an increasing capillary density. Also the number of SMCs increased, indicating the stabilizing role of these cells. Denuded ECs no longer inhibit SMC from migrating into the vessel lumen and as a consequence the SMCs narrow the blood vessels. Stoeckius and colleagues investigated the essential pathways in growth arrest of SMCs [120]. The observations provide important mechanistic insight into the molecular mechanisms underlying the transition of human vascular smooth muscle cells from proliferative to contractile phenotype and the role of bilirubin in this transition.

#### Patents on Endothelial Cells and Smooth Muscle Cells for Vascular Grafts

Patents using and affecting ECs or SMCs have been published nearly every year. The majority of patents aim the inhibition of SMC proliferation and migration. Therefore the injection of different pharmaceutical agents like peptides is one way to treat vascular diseases. Nabel patented a polynucleotide comprised of a thymidine-kinase gene, inhibiting SMC proliferation, whereas Suda developed an antibody against calcification of atherosclerotic plaques [121,122]. Wolinsky developed a catheter harboring balloons filled with heparin to prevent restenosis by inhibiting SMC to proliferate [123]. Altman patented a method for delivering therapeutic amounts of anti-restenotic agents [124]. Edelman and Uchida built matrices, which can be seeded with ECs or SMCs [125,126]. An adequate amount of ECs on the scaffold inhibits SMC proliferation. Takano developed an artificial vessel by coating collagen and fibronectin with SMCs, whereas Cao developed an apparatus to construct blood vessels *in vitro* [127,128]*.* Within this device ECS and SMCs are exposed to biomechanical factors to stimulate proliferation, directional arranging, and the production of extracellular matrix proteins. Other inventions aim the induction of endothelialization, e.g., by secreting agents to target EC migration. Burleigh patented a polypeptide to recruit ECs and the group of Jianhong, registered a scaffold containing amino acid sequences of collagen to capture ECs [129,130]. The scaffold maybe used as tissue engineering blood vessel rack, on which smooth muscle cell and/or endothelial cell may be planted. Iwazawa’s invention comprises a recombinant gelatin with an amino acid sequence derived from collagen [131]. Hoganson patented an *in vitro* model with a seeded (ECs and SMCs) scaffold to investigate the influence of pharmaceutical agents [132].

### 3.4. Other Primary Tissue Cells for Vascular Grafts

Although ECs and SMCs are the most prominent cells of the vasculature, other primary tissue cells have been investigated in view of their potential to promote vascular healing as well. Interesting candidates are cardiac precursor cells such as myoblasts, myofibroblasts, fibroblasts, and metakaryotic stem cells. While the obvious use of cardiomyocytes is more or less limited to the heart, fibroblasts are part of the blood vessel structure. Fibroblasts are of mesenchymal origin and present the main cells of connective tissue, synthesizing collagen and the extracellular matrix and thus the structure of the tissues. This makes them a suitable link in grafts between a biomaterial and the seeded tissue-specific cells. Kim and colleagues seeded among others dermal fibroblasts on decellularized human adipose-tissue-derived extracellular matrix sheets and observed their integration into the matrix [48]. Their results suggest that recellularized ECM sheets are a promising substitute for defective human tissues, including blood vessels. Kellar stimulated angiogenesis within a region of cardiac infarction used a scaffold-based 3D human dermal fibroblast culture as epicardial patch [133]. They observed higher numbers of arterioles, venules and capillaries in comparison to control groups and conclude the potential use for myocardial tissue repair. Tosun and his colleague seeded human myofibroblasts onto the abluminal site of *ex vivo* derived vascular collagen/hydrogel scaffolds and investigated different culture conditions [134]. The cells migrated towards the source of nutrients displayed by the perfused lumen of the decellularized vessel. Formigli and colleagues transplanted myoblasts into a chronic MI region of pigs and observed settlement of these cells in the ischemic scar and around blood vessels with an activated endothelium [135]. The invention of Gostjeva provides methods to identify molecules to modulate proliferation and/or migration of metakaryotic stem cell disorders, such as blood vessel wound healing disorders, including restenosis [85].

## 4. Summary of Cell Types for Vascular Grafts

The nearly unlimited supply of ESCs due to their self renewal capacity, in combination with their ability to differentiate into any cell type of the germ layer, makes this cell type a very interesting candidate for future approaches in regenerative medicine. However, their ability to expand indefinitely *in vitro* in cell culture gives also rise to spontaneous teratoma formation *in vivo*, displaying a non-negligible potential risk for patients. In addition, the use of ESC is limited in many countries due to ethical concerns related to the isolation of these cells from early embryos [2]. A promising other pluripotent stem cell source, iPS, was introduced in 2006 by Takahashi and Yamanaka [4,5]. This artificial cell type can be produced in large amount from various tissues which not only limits the ethical concerns, but also provides the possible use of autologous cells. Thus, it is not astonishing that applications of ESC in vascular healing approaches are decreasing, while approaches with iPS as cell type of choice are rising. Unfortunately, iPS have a similar risk to cause teratoma formation as ESCs, which is not dwindling substantially, because the reprogramming process is no longer done using retrovirus-derived vectors which have a carcinogenic potential on their own. The risk of causing teratoma is linked to the pluripotency of the cells and the fact that until now there is no protocol leading to ESC or iPS differentiation in 100% of the induced cells. Hence, adult stem cells with their lower plasticity are the focus of attention in most publications and patents linked with vascular healing (Figure 1). The most commonly used adult stem cells are mesenchymal stem cells obtained from bone marrow and adipose tissue. These cells can be differentiated into the desired cell types, namely endothelial and smooth muscle cells and expanded in cell culture to reasonable amounts for future applications in patients. Most researchers agree that immune reactions to this cell type are low and, anyway, they can be isolated from the patient itself if adipose tissue is the cell source. However, possible tumor formation, although less than in pluripotent stem cells, cannot be neglected. A new cell type with a lower potential caught the attention of scientists when it was discovered that most tissue contain precursor cells which were thought to be present only in tissue types with a high cell turn-over or a high regeneration potential. With the discovery of EPCs by Ashara and Shi, a new cell type for vascular research was at disposal that could be isolated from peripheral blood of a patient, thus causing no undesired immune reactions, and easily be differentiate into ECs and SMCs [57,58]. At the same time period, other stem and precursor cell types with variable plasticity were indentified and explored as well (see Section 3.4). Primary tissue-derived ECs and SMCs have been an early choice due to their natural role in the vasculature. But the limited availability of this cell type in humans due to the cell source being arteries and veins, the high risk of immune reason using allogenic approaches (if the cells are not derived from the umbilical cord), besides the very limited possibility for long-term culture and proliferation *in vitro* keeps the interest in this cell type to a steady but low level.

**Figure 2:**
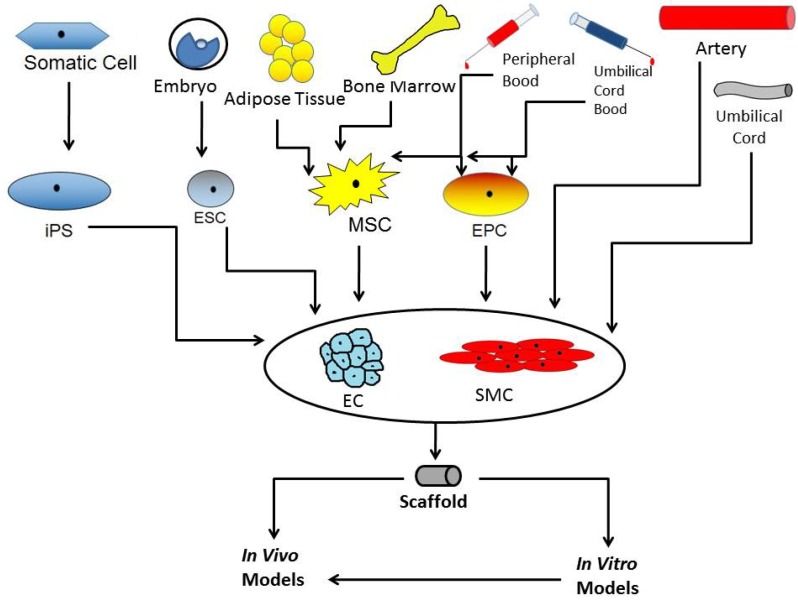
Cells used on grafts to promote vascular healing. The picture shows the different sources of cells used to promote vascular healing. Endothelial cells and smooth muscle cells can be obtained directly from primary tissue, e.g., arteries or umbilical cord or via differentiation of stem cells. Those stem cells can also be used directly on a graft. The cells can differentiate into the desired cell type and then be transplanted into animal models.

Taken together, the number of publications and patents using a huge variety of stem and precursor cells with distinct but divers plasticity for grafts promoting vascular healing is increasing. Figure 2 gives an overview of cell sources used to promote vascular healing. The success of such an approach is also dependant on the scaffold material which must be adapted not only to the respective tissue but also to the used cell type. Only a well-founded understanding of the metabolism of the respective cell type, combined with a tightly monitored and orchestrated procedure using the chosen well-tailored biomaterial might lead to a successful application of these grafts in human in the future.

## 5. Scaffolds for Stem Cell Engineering

The targeted control of the microenvironment for stem cell engineering plays a key role in tissue engineering of vascular grafts. The mimicking of the natural microenvironment by scaffold materials includes both biochemical and biophysical aspects. Whereas biochemical interactions of scaffold material and cells are dominated by the specific chemical structure of the scaffold surface, the biophysical requirements like matrix rigidity and matrix actuation can vary by several parameters, e.g., scaffold porosity and degradability. A broad spectrum of required properties was identified. The materials’ properties portfolio should support cell adherence, survival and proliferation, include biocompatibility, and—with respect to the desired function—higher or lower rates of biodegradability. Additionally, the mechanical and physical properties should match the same range as native tissues. Therefore, artificial three dimensional tubular scaffolds must exhibit a highly porous structure to allow cell adherence and proliferation. For vascular grafts, completely biological materials like cells on endothelial cell matrix (ECM) or bioartificial materials mostly consisting of synthetic polymeric scaffolds covered with cells are addressed in various studies and recently reviewed by Parizek and Chlupac [136,137].

Engler, Gilbert and Holst demonstrated the crucial role of mechanical properties, especially the elastic behavior and scaffold stiffness, though showing that the stiffness contributes significantly to the mesenchymal stem cell differentiation and the self-renewal [138,139,140].

### 5.1. Scaffolds Materials

**Figure 3:**
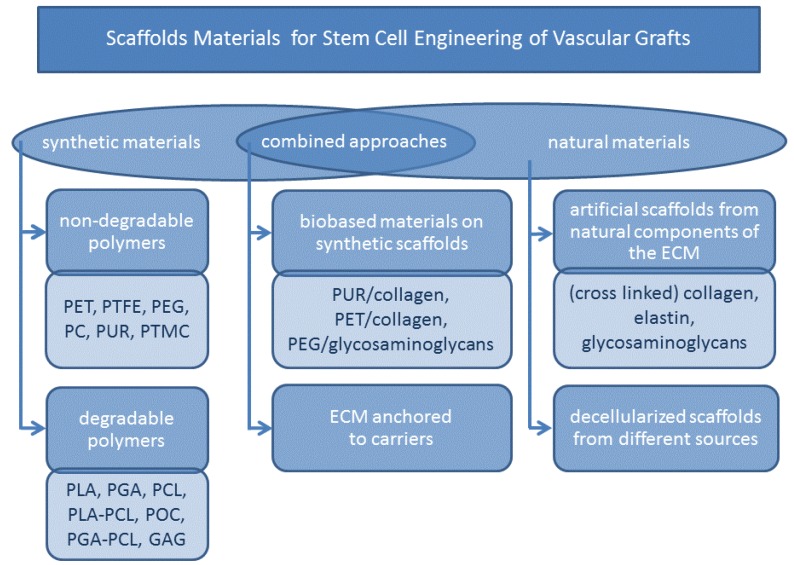
Scaffolds materials for stem cell engineering of vascular grafts. This scheme gives an overview about the materials discussed in this review with respect to the origin of the scaffold material. Synthetic and natural materials are addressed in the discussion as well as combined approaches to scaffold materials.

The development of tailor-made scaffolds for vascular healing shows promising results, and as a result, a tremendous number of papers and patents delve into the identification of suitable materials. In general, scaffold materials for stem cell engineering can be based on natural and synthetic polymers. Both groups can be subdivided into degradable and non-degradable materials (see Figure 3). Synthetic and natural materials are addressed as well as combined approaches. In the following, an overview will be given of the current state-of-the-art scaffold materials, including fabrication techniques and criteria for scaffold evaluation.

#### 5.1.1. Synthetic Polymers

The use of synthetic polymers allows a target-oriented design of desirable properties. The chemical structure, molecular mass, and its distribution can be controlled by the synthesis method of the polymer material.

(a) Non-degradable polymers

Non-degradable materials are mostly chosen to provide a scaffold with long-term stability. Materials like polyethylene terephtalate (PET), commercially produced as Dacron^®^, and polytetrafluoroethylene (PTFE), also known as Gore-Tex^®^, are candidates that dominated the first developments of artificial vascular grafts. The mechanical properties are incompatible with soft tissue regeneration, but are advantageous in composite scaffolds. A recently published study demonstrates the utilization of polyesters as carrier material for collagen. *In vivo* experiments were performed by Baguneid and colleagues to develop tissue-engineered small-diameter vascular bypass grafts [116]. Within the last two decades, other polymers have been studied including poly(ethylene glycol)s, polycarbonates, and polyurethanes. Poly(ethylene glycol) (PEG) is a hydrogel forming synthetic polymer. A large series of studies on vascular graft scaffolds focus on PEG and corresponding copolymers due to their ability to resist protein adsorption during *in vivo* application. Avoiding platelet adsorption is of high importance for the development of vascular implants. Benoit and colleagues studied the influence of small functional groups on controlled differentiation of hydrogel-encapsulated human mesenchymal stem cells [141]. PEG-based materials can be modified to adopt required biofunctionalities, for example with proteolytic degradable peptides, adhesion peptides, growth factors or collagenase sensitive peptide sequences [142]. Acrylated PEGs can be photopolymerized in direct contact with cells and tissues [143]. Polyurethanes can be synthesized using a broad variety of appropriate monomers. Based on the chemical structure of the monomers, polymers with adjustable properties and high biocompatibility can be prepared for medical applications. In terms of *in vivo* or in cell culture conditions, polyurethanes are mostly non-degradable polymers. However, using some polyesterols as building blocks for polyurethanes can introduce a certain degree of degradability. Hemocompatibility and biocompatibility can be combined with designable mechanical properties ranging from elastomer properties to stiff and rigid polyurethane plastics [144,145]. Within the last two decades, polyurethane materials have been investigated as scaffold materials for various medical applications as described in many review articles [136,142,146,147]. Owing to its three-dimensional polymer structure, polyurethanes can take on many different forms including foams, microspheres, microcapsules, nanocomposites, and membranes [148]. Additionally, polyurethanes can be combined with natural materials to generate composites (see paragraph (c) Composites). Another porous non-degradable polymer used for tissue engineering of small-diameter blood vessels is poly(1,3-trimethylene carbonate) (PTMC). In particular, PTCM with a molecular weight of M_n_ = 4 × 10^5^ g·mol^−1^ was cross-linked by gamma irradiation in an inert nitrogen atmosphere. The products are flexible elastic and creep resistant networks with 50%–70% gel content. Furthermore, PTMC is highly biocompatible, as determined by cell adhesion and proliferation studies using various relevant cell types [44].

(b) Degradable polymers

Biodegradability has already been successfully implemented in certain artificial systems and presents a major advantage for tissue engineering of replacement vessels. However, certain biodegradable polymers are still not fully reviewed in terms of long-term toxicology. Degradable materials provide the opportunity to adjust the required scaffold properties during treatment and allow for a subsequent replacement of the synthetic material by naturally produced tissue. A broad variety of polymers have been studied in detail, including polyglycolic acid (PGA), polylactic acid (PLA), and copolymers there of like PGA-PLA, polycaprolactone (PCL) and copolymers with polylactic acid (PLA-PCL), poly(1,8-octanediol-*co*-citric acid) (POC), corresponding citric acid-based biodegradable elastomers, and glycosaminoglycans (GAG).

For the evaluation of the use of an induced pluripotent stem cell sheet for the construction of tissue-engineered vascular grafts, PLA-based biodegradable tubular scaffolds were engrafted in congenital heart surgery. The transplantation of tissue engineered vascular graft was successful, safe, and effective in humans [26]. Co-polymers of polylactic acid and ε-caprolactone (PLA-PCL) are interesting biodegradable materials for vascular graft biodegradable tubular scaffolds. Bone marrow-derived mononuclear cells, differentiated smooth muscle cells, or endothelial cells were seeded onto PLA-PCL scaffolds. The results showed diverse cells living in the vascular drain exhibiting similar properties of native vessels [26]. Copolymers of polylactic acid, as well as polycaprolactone modified with polyglycolic acid, exhibited tensile mechanical properties more similar to those of dog inferior *vena cava* [54]. No sign of thrombosis, stenosis, or dilatation occurred up to eight weeks after implantation of vascular graft tissue-engineered polyglycolic acid modified PLA-PCL scaffolds cultivated with bone marrow derived mononuclear cells [53]. POC and citric acid-based biodegradable elastomers were used as hemocompatible and biodegradable elastomers for vascular tissue engineering. Once differentiated, endothelial cells’ phenotype and function on POC were assessed according to presence of the EC-specific markers von Willebrand factor, platelet EC adhesion molecule, and vascular endothelia cadherin, metabolism of acetylated low-density lipoprotein, secretion of the anti-thrombogenic factors nitric oxide and prostacyclin, inhibition of platelet adhesion and clotting processes *in vitro*. Porcine endothelial-like cells on POC showed phenotype, function, and clotting responses similar to those of primary aortic ECs [68].

(c) Composites: Biobased materials on synthetic scaffolds

The combination of synthetic and natural materials to form so-called hybrid scaffolds allows generating a wide range of parameters, adjustablein a manner that supports stem cell adhesion, differentiation and proliferation within a designed microenvironment [149]. Whereas the synthetic fraction of the scaffolds allows tailoring the physical and chemical properties of the material like mechanical behavior, wetability, and electrical properties, the control of cell behavior and biological activity can be achieved and varied by the bioactive components.

Polyurethane/Collagen: The combination of collagen and polyurethane is very promising for cell culture scaffolds. The enormous potential of such hybrid scaffolds is evident in the following examples: A cell mixture containing Flk1-positive cells (ca. 30%) derived from murine embryonic stem cells was added to a compliant microporous tube made of segmented polyurethane. The luminal surface of the tube was fully covered with the cells by pre-incubation for two days in the presence of vascular endothelial growth factor. After two days of additional incubation without VEGF under static conditions, layering of the grown cells, mostly smooth muscle actin-positive cells, was observed only on the luminal surface of the tube [9]. Endothelial progenitor cells derived from the peripheral veins of dogs were isolated by using a density gradient method. The cells were proliferated *in vitro* in EGM-2 culture medium, pre-lined on the luminal surface of *in situ* formed collagen type I meshes as an extracellular matrix, and wrapped with a segmented polyurethane thin film with multiple micropores as a compliant scaffold [77].

PET/Collagen: Another opportunity is a combination of collagen with a synthetic scaffold material like poly(ethylene terephtalate), known as Dacron^®^, or expanded polyethylene terephtalate (ePET), which are still widely used since their first introduction in 1957 [150]. These scaffolds provide relatively good mechanical properties, but must be modified at the surface due to their hydrophobic character in order to allow the reconstruction of an endothelial cell layer. Especially in small diameter vascular grafts, the limited cell adhesion can cause thrombogenic effects due to adhesion of thrombocytes [142]. Van Damme and colleagues studied Dacron^®^-based scaffolds combined with collagen or other protein-based natural components like albumin or gelatin [151]. A loss of structural integrity of the graft resulted in limited longevity of both the implants as well as the scaffolds. The possibility of fiber reinforcement of a collagen matrix, e.g., using PET fibers, provides the opportunity to control and tailor the mechanical strength of the resulting hybrid scaffolds. Successful proliferation of MSC in PET reinforced collagen sponges is recently reported by Takamoto [152].

PEG/Glycosaminoglycans: Glycosaminoglycans are also used in combination with synthetic polymers, e.g., poly(ethylene glycol) for scaffold hybrid fabrication. Serving as a reservoir for growth factors, the glycosaminoglycan fraction in such hybrids allows a controlled delivery of the released factors. Sustained delivery of SDF-1α from heparin-based hydrogels was studied by Prokoph and colleagues to attract circulating pro-angiogenic cells [153]. In detail, the influence of released SDF-1α on the surveillance of migration of endothelial progenitor cells was investigated.

#### 5.1.2. Natural Polymers

Scaffolds based on natural polymers can be subdivided into (a) Decellularized scaffolds from different sources and (b) Artificial scaffolds from natural material components of the ECM. Many *in vitro* and *in vivo* studies are available that describe the use of biopolymers extracted from animal tissues. With respect to the imitation of extracellular microenvironments by the components of the ECM like collagen [118], elastin [154], and combinations thereof [155], a large variety of materials are studied regarding their influence on stem cell differentiation. Native tissues can be decellularized to obtain a scaffold material which reflects the structural characteristics of the natural microenvironment of the desired cells, as well as their chemical composition regarding the corresponding building blocks like collagen, elastin, or glycosaminoglucan [155]. Recently, a novel *in vitro* approach for anchoring ECM produced by cells to the surface of cultural carriers is described by Prewitz and colleagues [156]. This approach suggests that human bone marrow derived MSCs cultured on ECM modified carriers show proliferation rates three times higher than those under comparable cultural conditions. Since the microenvironment of such ECM-derived material mimics natural conditions, these experiments demonstrate impressively how to understand the control of stem cell differentiation and proliferation by biomolecular and physical signals of stem cell niches.

Whereas the production of ECM under *in vitro* conditions requires complex and expensive techniques, collagen is available inexpensively in technical scale [157]. Collagen is widely used for artificial blood vessels and scaffolds prepared from fractions of the connecting tissue in *tunica intima* and *tunica media* [147]. The main advantages of collagen stem from the unique properties of this natural polymer, such as a low immunogenicity and good cell adherence properties, e.g., for endothelial cells [57]. There are two general options in using collagen: decellularized animal blood vessels and artificially designed scaffolds made from collagen hydrogels. Collagen hydrogels are well-suited as material for artificial scaffolds, since they are available in high qualities for medical applications. Successful collagen use as scaffold material for *ex vivo* differentiation of endothelial and smooth muscle cells from human cord blood progenitors in three-dimensional culture is reported by Le Ricousse-Roussanne [158]. Further interesting materials for cell culture include collagen sponges derived from freeze-drying processes, as described by Bohrer and colleagues [159]. Collagen sponge material is commercially available in several qualities for applications such as periodontal regeneration or wound healing [160,161].

Collagen is a suitable candidate to mimic important features of the stem cell niche environments, but does not provide enough mechanical strength required for vascular graft implants. As a result, many studies try to overcome this deficiency. One way to improve the biomechanical properties of collagen is the 3D network formation via crosslinking, applied either to a hydrogel or a structured collagen sponge-like matrix. Early studies from Chvapil and co-worker demonstrated that the sponge-like form allows cells to adhere and to proliferate within the pores of the sponge [162]. However, in many of the references about collagen-based scaffolds, no detailed information is given about the origin and the pretreatment of the collagen, though this information is crucial for applications of collagen in stem cell engineering.

Glycosaminoglycans are polydisperse mixtures of polysaccharide chains of varying lengths with an average molecular weight of 10^4^–10^6^ g/mol and a natural constituent of animal cells [163]. GAGs bind to proteins primarily through the interaction of its functional groups. Mainly the sulfo and carboxyl groups of the highly charged polyanions enable the interaction with a widespread variety of proteins [164,165]. The binding of heparin to antithrombin is well-known [166,167]. Specific oligosaccharide subsets within GAGs are the interacting parts with growth promoting proteins such as vascular endothelial growth factor (VEGF) [168].

Total synthetic approaches require simplification of the complicated GAG structure and ambitious multistep synthetic routes to control the structural setting and the stereochemistry [62,169,170,171]. Significant improvement of the synthetic approach was made by learning from nature about the role of enzymes in biosynthesis. The preparation of natural and artificial GAG analogues succeeded in applying three enzyme families: GAG lyases, synthases and sulfotransferases [172]. Today, glycosaminoglycans studies are driven by the evidence for a special ability to support the maintenance of vascular smooth muscle cells in quiescent and contractible manner [136,172].

### 5.2. Scaffold Fabrication Methods

#### 5.2.1. Conventional and Nano-based Fabrication Methods

Conventional methods to generate polymer-based scaffolds include various moulding and casting processes, spinning, sintering, and extrusion techniques. The fabrication of three-dimensional scaffolds is possible by generating pores via particle or other selective leaching methods, phase separation and different gas forming methods. Polymer fibers can be treated via textile formation processes such as braiding, weaving, and knitting. Polymeric scaffolds have been investigated in form of foams, sponges, gels, or hydrogels. Additionally, scaffold surfaces are modified to deliver biologically active agents such as tissue growth factors such as VGEF, as reviewed in detail by Sachloz [173] and Moroni [174].

Gels and hydrogels are used for medical applications, particularly in drug-delivery. Within the last decade, hydrogels have also been studied as scaffold materials providing the possibility to encapsulate cells. Thus, a number of materials have been investigated including non-resorbable polymers, polyesters and polyamides, biodegradable polymers based on collagen, glycolic acid, lactic acid or hyaluronic acid. Natural biopolymers such as polysaccharides (e.g., hyaluronic acid derivative), synthetic polymers (e.g., poly(hydroxyethyl methacrylate)) or semi-synthetic derivatives (e.g., collagen-PLA composites) are able to form hydrogel structures. The corresponding three dimensional polymer networks can be realized via radical or photopolymerization, induced by ultraviolet irradiation or self-assembling peptide hydrogel structures supporting differentiation and transdifferentiation of cells. Stem or progenitor cells are encapsulated within these self-assembling peptide hydrogel structures. Thus, peptide-based hydrogels are developed and functionalized with growth factors or ECM components for controlled drug release, which enables the cells to differentiate or transdifferentiate within the structures [175]. The limited mechanical and viscoelastic properties of hydrogels requires a combination with more stable 3D structures for scaffold application in tissue engineering [176].

Polymeric foams are widely used in medicine as scaffolds and drug release matrices. Polymer surfaces can be textured as a result of foaming processes. This is of vital interest since surface morphology and roughness have been demonstrated to influence the physiological response of cells such as cell attachment, morphology, and differentiation [177]. Porous foams based on racemic poly(lactide-*co*-glycolide) copolymers and microcellular foams made from biodegradable or non-biodegradable polymers with pores throughout the material having a diameter of about 1 to 200 microns are investigated and approved for human clinical use [178].

Nanomaterials: The discovery of fullerenes (in 1985) and carbon nanotubes (in 1991) led to a tremendous development of novel nanomaterials. Additionally, investigations ensued regarding their use in medical applications [179,180,181]. Carbon nanotubes possess exceptional mechanical, thermal, and electrical properties, which facilitates their use as reinforcements or additives in various biomaterials. This particularly improves their mechanical behavior. Carbon nanotubes are synthesized and added to conventional polymer scaffolds to promote and guide tissue growth and regeneration. Today, a broad variety of nanostructured materials are developed such as particles, spheres, tubes, fibers, and dendrimers [182,183,184]. Additionally, nanocomposites, nanosurfaces, and nanosphere-immobilized biomaterials have gained increasing interest in regenerative medicine due to their ability to mimic the extracellular matrix (ECM). Nanomaterials have been intensively studied in the last decade for their use in tissue engineering. Material synthesis, characterization and *in vitro* analysis of nano-based scaffolds for tissue engineering applications are reviewed by Wang and co-workers [185].

Nanotechniques developed in the past decade include electrospinning and various rapid prototyping methods used for 3D scaffold design [186,187,188]. Three dimensional printing, fused deposition modelling (FDM), stereolithography (SL), selective laser ablation (SLA), and selective laser sintering (SLS) are considered to be the most promising techniques for smart scaffold fabrication delivering tailored three-dimensional scaffold architectures and nanostructured surfaces [174,189,190]. In particular, scaffolds with a controllable interconnected pore network are available via rapid prototyping technologies, which allows for improved cell migration and nutrient exchange. Electrospinning provides fibrous scaffolds, mimicking the dimension and topology of ECM fibers. Filaments can be formed on the nanometer scale used as medical membranes and scaffolds for tissue engineering. Sitharaman and co-worker reported the preparation and investigation of porous 3D printed scaffolds based on functionalized polyesters for tissue engineering [191]. Applications in tissue engineering are mainly focused on utilization of nanomaterials to improve mechanical properties of the scaffolds. Nanofibers are prepared via electrospinning, phase separation, or self-assembling techniques. Biodegradable polymer nanofibers mimic the nanofibrillar structure of ECM. The nanoscaled collagen fibrillar structure (50–500 nm in diameter) has been found to enhance cell-matrix-interactions [187]. Lim studied micropatterning by femtosecond laser ablation of electrospun nanofiber tissue scaffolds based on composites of poly(caprolactone) and gelatin [192]. Nanostructured mesoporous silicon can be used for discriminating *in vitro* calcification of electrospun scaffold composites [193]. Another approach combines freeze-drying and foaming techniques to generate highly porous 3D scaffolds [194].

#### 5.2.2. Network Formation via Polymer Cross-Linking

In 1998, Gagnieu registered a patent for cross-linking techniques of collagen derivatives and their utilization as biomaterials [195]. A decade later, Zeugolis and co-workers reported a study of a wide range of cross-linking approaches, including chemical, physical, and biological methods regarding the influence of cross-links on the properties of resulting collagen structures [196]. Structural evaluation revealed a closely packed inter-fiber structure independent of the cross-linking method employed. Thermal properties were dependent on the cross-linking method and closely matched native tissues. Many efforts are being made to improve the preparation of three-dimensional networks used as scaffolds for tissue engineering. However, the generation of tailor-made 3D polymer networks with well-defined structure and adjusted chemical and physical properties is still a challenge for material scientists. Beside crucial requirements for scaffold materials, there are further special needs dependent on the specific application, e.g., the mechanical scrape strain of polymers used in wound assay models and the chemical resistance in special cell culture conditions [110,197]. The creation of a discrete polymer network would be a possible approach to influence those properties.

In terms of their 3D structure, collagens are physically cross-linked co-polymers. Dipole forces and hydrogen bonds cause a natural helical network. The formation of natural cross-links is based on enzymatic reactions by lysyloxidase [198,199]. In addition, further intra- and intermolecular cross-links can be developed [200]. Intramolecular diagonal cross-links are formed by spontaneous reaction of adjacent aldehyde groups via aldol condensation reactions [201]. In contrast, intermolecular cross-links are formed by reactions of the ε-amino groups of certain amino acids (e.g., lysine or hydroxyl lysine).

Besides the naturally occurring covalent cross-linking, artificial chemically induced cross-linking can be performed to modify collagens for cell culture experiments. When collagen comes into contact with water, the water is bound either as hydrate or capillary water. Excessive bulking of collagen layers in water leads to destructions of the compounds [202,203,204]. To improve the resistance against humidity, chemical modification of side groups (e.g., ε-amino groups of lysine and hydroxyl lysine, carboxyl groups of asparagine and glutamine acid) are performed resulting in cross-linked networks. In the following paragraphs, the most frequently used cross-linking methods are discussed more in detail: Cross-linking via chemical reagents or enzymes, via radiation (UV, gamma or electron beam), or thermal treatment.

**Figure 4:**
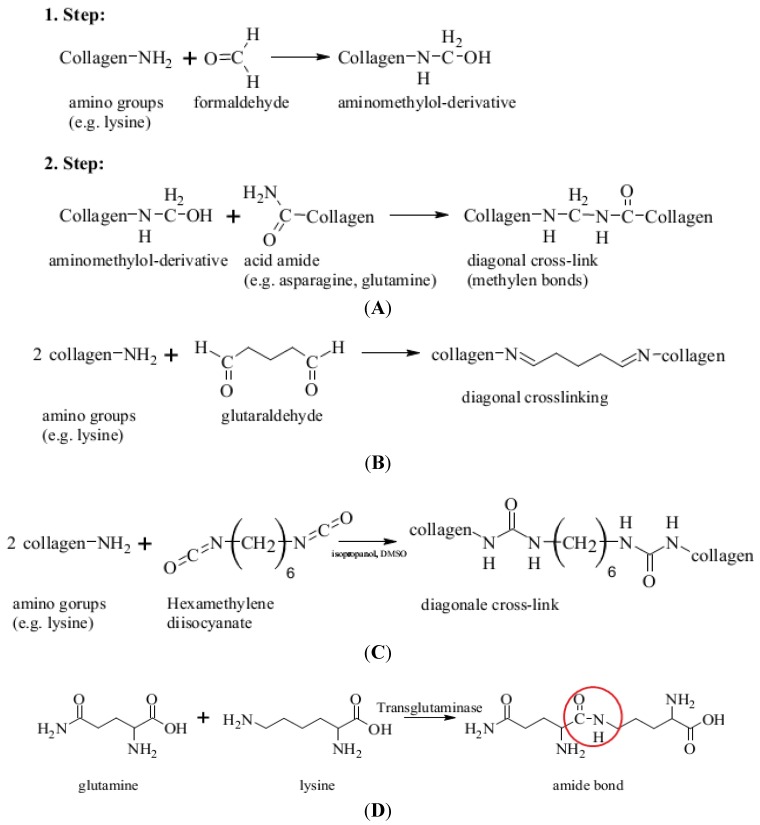
Cross-linking reactions. (**A**) Cross-linking via formaldehyde. In the first step amino groups of collagen (e.g., in lysine entities) react with formaldehyde to form an aminomethylol-derivative. In the second step, a diagonal cross-link is formed by reaction of the aminomethylol-derivative with an acid amide; (**B**) Cross-linking via glutaraldehyde. Reaction of amino groups of two collagen molecules (e.g., in lysine entities) with glutaraldehyde leads to diagonal crosslinking of collagen molecules; (**C**) Cross-linking via hexamethylene diisocyanate. Hexamethylene diisocyanate can react with amino groups to crosslink collagens; (**D**) Cross-linking via enzymes. During enzymatic cross-linking with transglutaminase an amide bond is generated via conversion of glutamine and lysine units.

Crosslinking by chemical reagents: The majority of cross-linking reactions are performed via amino and amide groups. In rare cases, guanidine groups participate in the formation of a cross-linking bond [205,206,207,208,209,210]. Alternatively, bonds are formed between free carboxylic groups—here the reagent is not embedded in the collagen matrix reagent [211]. The reaction mechanism of the cross-linking reaction initiated by formaldehyde is shown in Figure 4A [200,212]. This formaldehyde-protein-reaction depends strongly on the experimental conditions and is not specific to lysine [212]. 

Another cross-linking mechanism involving two free amino groups and glutaraldehyde is shown in Figure 4B [213]. Unfortunately, this cross-linking reaction mechanism may cause subsequent hydrolysis reactions. Analogue to the mechanism shown in Figure 4A, hexamethylene diisocyanate leads to cross-linking by reacting with two amino groups [214]. The formation of amid bonds between free carboxyl- and amino groups is shown in Figure 4C. In addition, cross-linking can be reached via acyl-azides or aqueous solution of 1-ethyl-3-(3-dimethylaminopropyl)carbodiimide [211,215]. Formaldehyde and glutaraldehyde are the most commonly used compounds. Glutaraldehyde shows a higher activity with increasing pH values, finding a maximum at pH 8 [216]. In contrast, formaldehyde exhibits an increased activity at lower pH values (<6.5), an essential advantage considering that most collagens exhibit pH value in the range between 2.5 and 3.0. Decreasing cross-linking velocity could result in unstable, partially reversible compounds [200,206,207]. Cross-linking of glutaraldehyde is not only fast, the resulting products are also significantly more stable. Similar network qualities in terms of mechanical stability are found in collagen fibers treated with hexamethylene diisocyanate [216]. The cytotoxicity is lower for the acyl-azid method in comparison to the glutaraldehyde method. The use of 1-ethyl-3-(3-dimethylaminopropyl)carbodiimide with supporting agent N-hydroxysuccinimide (NHS) is a cell compatible cross-linking method. NHS takes part in the activation and not in the cross-linking. This discloses cytotoxic effects. The so improved collagen scaffolds are primarily used for cell culturing [214]. The relatively fast activation is not applicable in aqueous solutions, commonly used to prepare films and scaffolds [196]. Economic and technical reasons hamper the application of 1-ethyl-3-(3-dimethylaminopropyl)carbodiimide for cross-linking reactions [217]. Marzec and Pietrucha studied collagen cross-linking with hyaluronic acid (HA) to be used for scaffold fabrication [218]. Resulting networks were investigated by the dielectric spectroscopy over a wide range of temperatures and provided detailed information on molecular interactions in cross-linked collagen. Cross-linking and grafting of the collagen film by HA is effective not only in slowing down the biodegradation rate, but also in optimizing the dielectric properties of these systems.

Crosslinking by enzymes is a very efficient, nontoxic method to stabilize collagen matrices. The most common enzyme for cross-linking reactions is transglutaminase (TGM), which is commercially used in food industry to improve the texture, emulsion properties, firmness, and elasticity of protein containing food like yogurt, fish, or meat [219]. Water resistance and tensile strength of 3D networks can be improved using TGM [220]. In addition, TGM is used for modification of galantine [221]. Stachel *et al*. describes the improvement of biocompatibility of medical implant materials in mammalian host bodies by TGM cross-linking of raw collagen [219]. Claims are made regarding improvements in cell adhesion, proliferation, and differentiation. The transglutaminase catalyzes an acyl-transfer reaction between a γ-carbamide group of a protein bonded glutamine and an ε-amino group of a protein bonded lysine resulting in the formation of intermolecular or intramolecular ε-(γ-glutamyl)lysine isopeptide bonds as shown in Figure 4B [219,222]. However, disadvantages of this treatment are rather long reaction times (between 6 to 8 h) and the complex inactivation.

Crosslinking by irradiation: The different beam induced cross-linking methods have clear advantages towards chemical cross-linking methods. First, there is no need for additional chemicals or supporting agents. The combination of cross-linking and simultaneous sterilization makes these methods especially interesting for medical and biological applications. To realize beam sterilization, a minimum dose of 25 kGy is needed [223]. Thus, the collagen composition to be cross-linked and the radiation dose must be scaled and adjusted. High intensities can cause fragmentation and denaturation [224]. In general, three different types of irradiation treatment of collagen are established: UV radiation, gamma ray treatment, and electron beam radiation. The treatment with UV rays is based on the creation of free radicals in aromatic residuals of tyrosine and phenylalanine. Cross-linking reactions occur between those radicals limited by the rather low amount of aromatic amino acids in collagen [224]. Collagen consists of specific chemical units, which show absorption in the near (315–380 nm, UV-A) and middle UV range (280–315 nm, UV-B), whereas the aromatic amino acids absorb in the far UV range (280–200 nm, UV-C). Treatment times between 15 and 30 min are reported. The effective radiation depth is limited to a few nanometers and therefore to the immediate surface [225]. Photochemical cross-linking with riboflavin was investigated for the treatment of keratoconus, a degenerative disorder of the eye in which structural changes of the cornea cause a decrease in thickness [226]. Very recently, Hayes and colleagues published the effect of riboflavin/UV collagen cross-linking therapy on the structure and hydrodynamic behavior of the ungulate and rabbit corneal stroma [227].

Gamma ray treatment (^60^Co-source) of collagen leads to cross-linking by imine and aldol bonds, which are generated by oxidative deamination. In addition, covalent links are formed from emerging free radicals [195,228].

**Figure 5:**
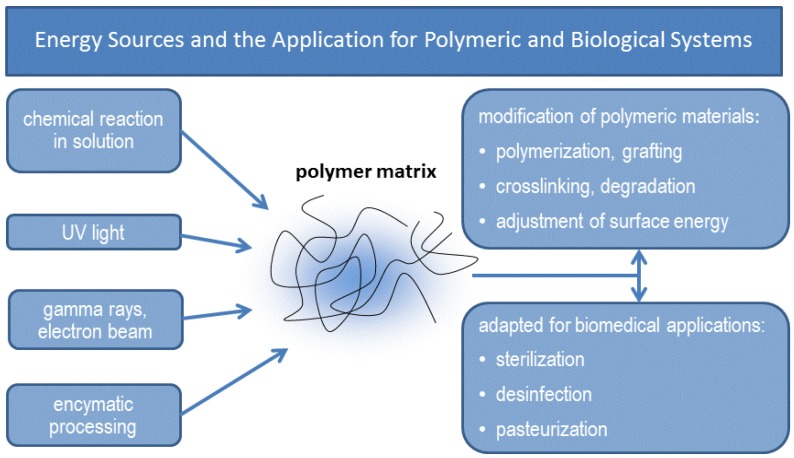
Energy sources and their application for polymeric and biological systems. Chemical reaction, UV-light, gamma rays, electron beam or enzymatic processing are valuable tools for the modification of polymeric materials. Hereby, miscellaneous, partially contrary effects are observed including cross-linking reactions. For biological applications, sterilization, disinfection and pasteurizing effects are described.

Crosslinking by Electron Beam Radiation represents an innovative method for treatment and sterilization of biomaterials. In contrast to gamma rays (electromagnetic waves), electron beam provides a particle radiation [228]. In general, the effect of electron beam irradiation on matter is rather complex. Given that radicals are produced, this method can be used for the modification of polymers, particularly polymer surfaces and treatment of biological materials, summarized in Figure 5. Besides cross-linking and degradation, which are in most cases the desired outcomes of electron beam treatment, further processes can take place depending on the chemical feature of the polymer, involved process gases, and other energy depending factors [229,230]. Scission of the peptide bonds in collagen occurs during electron beam treatment under standardized conditions using inert gas atmosphere. Although electron beam treatment can lead to instability of the helical structure, the helix can be maintained if the links are as close as possible to the scission position. This effect is observable when high radiation doses and temperature above the melting temperature of collagen in a nitrogen atmosphere are used, resulting in intermolecular covalent cross-linking. The cross-linking density increases with increasing radiation dose [231]. The disadvantage in comparison to the gamma ray treatment is the lower depth of a few μm up to a few mm [223]. However, this is overbalanced by the advantages of a disengageable radiation source, short treatment times of few minutes, an easy adaption of treatment parameters, and a lower impairment of biomechanical properties of the corresponding biological network [232]. Besides the irradiation energy, an important parameter is the dose absorbed by the material. The wide range of applications of electron beam treatment is shown in Table 1.

**Table 1:** Selected industrial applications of irradiation processing [233,234,235,236].

Irradiation target	Net effect	Dose range (kGy)
Food	Cold pasteurization	0.3–30
Medical disposable items	Sterilization	10–60
Cellulose and derivates	Degradation	5–50
Polymer coatings	Curing	30–160
Polyolefin foams	Crosslinking	40–80
Heat-shrinkable materials	Memory imparted	75–250
Soft materials (especially rubber)	Cross-linking/Vulcanization	80–400
Fluoropolymers	Degradation	500–1500
Gemstones	Coloration	>10,000

The use of electron beam technology for the modification of polymeric surfaces or for the linking of surface modified layers to organic support structures is represented in several examples [237,238]. However, systematic investigations of the electron beam treatment in modifying biological polymer materials like collagen are rarely published in a well-defined manner. The predominant process in the application of electron beam treatment of collagen-based substrates is described as radically initiated cross-linking [231]. Investigations regarding the detailed mechanism of the cross-linking reaction are not yet available. One can assume that network formation originates in radical creation in peptide-based structures, which is sufficiently described for UV- and gamma ray treatment [195,206,239,240,241]. Furthermore, bond scissions are observed in addition to cross-linking [242]. Investigations of co-cultures of hepatocytes and endothelial cells on electron beam structured poly(N-isopropylacrylamide) substrates are published [243]. Degradation is described as predominantly for cellulose based biopolymers [244,245]. On the other hand, natural rubber (Guttapercha, poly(1,4-*cis*-isoprene) and synthetic poly(1,4-*cis*-isoprene)) are well cross-linkable to achieve elastomers using electron beam treatment without cross-linking agents [244]. The electron beam treatment of biological polymers and co-polymers based on PLA and PHB can either achieve cross-linking or degradation, depending on the reagent [233,245,246,247,248,249,250,251,252]. However, detailed knowledge concerning the effect of electron beam treatment on the structure and properties of the treated polymers is predominantly known from investigation of synthetic polymers [234,235,244,246]. Polymers with either secondary or allylic bound carbon atoms in their polymer chain, like polyethylene, polybutadiene, polyisoprene, or silicones, tend to undergo cross-linking reactions. In contrast, polymers with tertiary carbons atoms in the polymer chain, like polypropylene, polymethylacrylate, polyisobutylene, polytetrafluoroethylene, or cellulose derivates, tend to undergo degradation by chain scission. When cross-linking dominates in contrast to bond scissions, the degree of cross-linking points is proportional to the absorbed dose and can be evaluated using relations between the degree of cross-linking and physical or mechanical properties [236,253]. However, it should be considered that the density of radicals is lower in comparison to chemical cross-linking reactions. The relative absorbed dose is commonly described in dependency of the area density of the polymer [254]. In summary, electron beam irradiation has considerable advantages compared to chemical based methods, beam induced UV, and gamma ray treatments including variable effective depth, short treatment times, disengageable radiation source, low heat emission, applicability at room temperature, no need for cross-linking agents, and sterilization effect [255,256]. This treatment method is well-established in medical engineering. A subsequent sterilization of the modified polymer substrate using steam pressure sterilization or ethylene oxide is not necessary. Thus, the technologically versatile process of electron beam treatment has the potential for innovative modification of cell culture materials, and fulfills the need for a simple commercial method with a limited number of procedural steps for modification and sterilization [256,257].

Cross-linking by thermal treatment is mainly realized via the so-called dehydrothermal method, which entails eliminating water in a stepwise process under vacuum in temperatures around 100 °C. Subsequently, it has been observed that a reformation of amid bonds via reaction of carboxyl and amino groups can lead to a cross-linked collagen matrix. Hiraok and colleagues investigated collagen sponges mechanically reinforced through the incorporation of poly(glycolic acid) fiber [258]. They prepared collagen-PGA dispersions that were freeze-dried, followed by dehydrothermal cross-linking to obtain collagen sponges incorporating PGA fiber to various extents. By scanning electron microscopy observation, the collagen sponges exhibited isotropic and interconnected pore structures with an average size of 180 μm, irrespective of PGA fiber incorporation. As expected, PGA fiber incorporation enabled the collagen sponges to significantly enhance their compression strength.

## 6. Summary of Scaffolds for Vascular Grafts

The target goal of tissue engineered vascular grafts and conduit vessels are systems that perfectly mimic natural vessels, while avoiding immunological rejection. Tremendous efforts are made to improve scaffold materials in terms of architecture, topography, and bioactivity. Primary attention is given to produce appropriate three-dimensional porous networks using conventional or nano-based fabrication methods. Regarding conventional methods, chemical, physical, and biological cross-linking methods can be used to generate scaffolds that closely match native tissues. In particular, the technologically versatile process of cross-linking via electron beam treatment has the potential for innovative modification of cell culture materials, and fulfills the need for a simple commercial method with a limited number of procedural steps for modification and sterilization. High expectations exist for the development of artificial vessel replacements using nanomaterials and rapid prototyping fabrication technologies. Most prominent candidates are electrospinning and laser printing technologies. Nanotechnology can provide an alternative platform to develop materials of higher mechanical strength, enhanced functionality, and resorbability for improving the quality of tissue engineered vascular grafts [259,260]. The future of new tissue engineered biomaterials depends on an enhanced knowledge and better understanding of both the structure-property relationships of the targeted scaffolds, and the most significant inter- and intramolecular, cellular, and tissue interactions. Tailor-made and appropriately characterized scaffold materials including hierarchically structured 3D networks and hybrid materials with enhanced bulk and surface properties will become a major preoccupation for successful scaffold development [156]. In particular, biomimetic and bioinspired multifunctional composite and hybrid materials with well-defined nanostructure and nanotopography that elicit the desired cellular and tissue response will be the primary task for the next generation of artificial substitutes. In addition to potential clinical applications, such engineered biomaterials will add fundamental contributions to basic research, including better understanding of the interactions of materials with living cell systems [260].

## 7. Conclusions

Cardiovascular diseases are the main cause of death in industrialized countries and can be expected to increase in the future due to an increase life expectancy of the population combined with a sedentary lifestyle and an increase in adipositas. This leads to the increasing need for treatment methods for vascular diseases, starting with lifestyle changes and drug treatments, to new methods such as tissue replacement strategies. Within the last decade, high hopes rely increasingly on the development of artificial vascular grafts which have shown major improvements due to innovations, both in cell culture and material science. Numerous new approaches have been published and patented using combinations of stem cell-based tissues on tailor-made biomaterials tested in small and even large animal models already, thus passing to some extent the preclinical phase. However, none of them have entered clinical trials phases until now.

In spite of these promising developments, tissue engineering of vascular grafts still has major problems to solve. The potential success strongly depends on the availability of an appropriate cell type for the graft material. All possible kinds of cells tested at the moment range from fully differentiated vascular tissue cells to stem cells with the full range of plasticity, indicating that the best solution has to be found yet.

Scaffold design for cell engineering is targeted to mimic the native cell microenvironment. Thus, a combination of biological, biochemical, physical, and mechanical interactions has to be adjusted. Within the last two decades, a widespread variety of scaffold materials and fabrication methods have been addressed, including natural and synthetic materials. Thus, both types allow the use of technically well-established fabrication methods and provide scaffolds with the desired mechanical properties. However, the resulting scaffolds, in particular the scaffold surface, must be modified and bio-activated to mimic the biochemical microenvironment of cells. Natural materials exhibit a closer analogy to the biochemical microenvironment. However, their use requires highly sophisticated production methods and, if processed with technical fabrication methods, additional crosslinking or reinforcement to gain scaffolds of sufficient mechanical stability. The use of cell expressed ECM and its immobilization on carriers comes as close as possible to the natural microenvironments and is therefore a promising path towards a well-founded understanding of the metabolism of the respective cell type. In the future, a successful application of vascular grafts in humans will combine tightly monitored and orchestrated procedures with well-tailored biomaterial.

## Abbreviations

AP: alkaline phosphatase
ATSC: adipose-tissue derived stem cell
3D: three-dimensional
Ac-LDL: acetylated-low density protein
Ang-1: angiopoietin-1
ASC: adult stem cell
α-SMA: alpha-smooth muscle actin
bFGF: basic fibroblast growth factor
BM-EPC: bone marrow-derived progenitor cell
BM-MSC: bone marrow-derived mesenchymal stem cell
BrdU: bromedesoxyuridine
CD: cluster of determinants
DMSO: dimethylsulfoxid
EC: endothelial cell
EGM-2: endothelial growth medium-2
eNOS: endothelial nitric monoxide synthase
EPC: endothelial progenitor cell
ePET: expanded poly(ethylene terephtalate)
ESC: embryonic stem cell
FACS: fluorescent activated cell sorting
FDM: fused deposition modeling
GAG: glycosaminoglycan
HA: hyaluronic acid
hATSC: human adipose tissue-derived cell
HC: heavy chain
HCAEC: human coronary artery endothelial cell
hCASMC: human coronary artery smooth muscle cell
hEC: human endothelial cell
hEPC: human endothelial progenitor cell
hESC: human embryonic stem cell
HGF: hepatocyte growth factor
HGF: hematopoietic growth factor
HLA-DR: human leukocyte antigen
hMSC: human mesenchymal stem cell
hSMC: human smooth muscle cell
hsp: heat shock protein
HSVSMC: human saphenous vein smooth muscle cell
HUASMC: human umbilical artery smooth muscle cell
HUVEC: human umbilical vein endothelial cell
HUVSMC: human umbilical vein smooth muscle cell
IGF-1: insulin-like growth factor-1
iPS: induced pluripotent stem cell
KDR/Flk-1: kinase insert domian receptor/fetal liver kinase-1
MI: moycardium infarction
MSC: mesenchymal stem cell
NHS: N-hydroxysuccinimide
PB-EPC: peripheral blood-derived endothelial progenitor cell
PB-MSC: peripheral blood derived mesenchymal stem cell
PCL: poly(caprolactone)
PDGF: platelet-derived growth factor
PECAM-1: platelet endothelial cell adhesion molecule-1
PEG: poly(ethylene glycol)
PET: poly(ethylene terephtalate)
PGA: polyglycolic acid
PHB: poly(hydroxybutyrate)
PLA: polylactic acid
PLA-PGA: poly(lactic glycolic acid)
PLA-PCL: poly(lactic acid)caprolactone
POC: poly(1,8-octanediol-*co*-citric acid)
PTHrP: parathyroid hormone-related protein
PTFE: poly(tetrafluoroethylene)
PTMC: poly(1,3-trimethylene carbonate)
PU: polyurethanes
NG2: nerval/glial antigen 2
SDF: stromal cell-derived factor
SL: stereolithography
SLA: selective laser ablation
SLS: selective laser sintering
SM: smooth muscle
SMC: smooth muscle cell
SMPC: smooth muscle progenitor cell
TEBV: tissue-engineered blood vessel
TEGV: tissue-engineered vascular graft
TGF-β: transforming growth factor-beta
TGM: transglutaminase
UB-EPC: umbilical cord blood endothelial progenitor cell
UC-EPC: umbilical cord endothelial progenitor cell
UV: ultraviolet
VCAM: vascular cell adhesion molecule
VE-cadherin: vascular endothelial cadherin
VEGF: vascular endothelial growth factor

## References

[1] Limbach C.A., Lange M., Schulze M., Tobiasch E. (2012). Recent patents on biomedical applications for the treatment of atherosclerosis. Recent Pat. Regen. Med..

[2] Donovan P.J., Gearhart J. (2001). The end of the beginning for pluripotent stem cells. Nature.

[3] Liu S. (2008). iPS cells: A more critical review. Stem Cells Dev..

[4] Takahashi K., Yamanaka S. (2006). Induction of pluripotent stem cells from mouse embryonic and adult fibroblast cultures by defined factors. Cell.

[5] Takahashi K., Okita K., Nakagawa M., Yamanaka S. (2007). Induction of pluripotent stem cells from fibroblast cultures. Nat. Protoc..

[6] Schulze M., Tobiasch E., Scheper T., Kaspar C., Pörtner R., Witte F. (2011). Artificial Scaffolds and Mesenchymal Stem Cells for Hard Tissues. Tissue Engineering III: Cell—Surface Interactions for Tissue Culture.

[7] Jin H.J., Bae Y.K., Kim M., Kwon S.J., Jeon H.B., Choi S.J., Kim S.W., Yang Y.S., Oh W., Chang J.W. (2013). Comparative analysis of human mesenchymal stem cells from bone marrow adipose tissue, and umbilical cord blood as source of cell therapy. Int. J. Mol. Sci..

[8] Calloni R., Cordero E.A., Henriques J.A., Bonatto D. (2013). Reviewing and updating the major molecular markers for stem cells. Stem Cells Dev..

[9] Shamblott M.J., Axelman J., Littlefield J.W., Blumenthal P.D., Huggins G.R., Cui Y. (2001). Human embryonic germ cell derivatives express a broad range of developmentally distinct markers and proliferate extensively *in vitro*. Proc. Natl. Acad. Sci. USA.

[10] Huang H., Nakayama Y., Qin K., Yamamoto K., Ando J., Yamashita J., Itoh H., Kanda K., Yaku H., Okamoto Y. (2005). Differentiation from embryonic stem cells to vascular wall cells under *in vitro* pulsatile flow loading. J. Artif. Organs.

[11] Nakagami H., Nakagawa N., Takeya Y., Kashiwagi K., Ishida C., Hayashi S., Aoki M., Matsumoto K., Nakamura T., Ogihara T. (2006). Model of vasculogenesis from embryonic stem cells for vascular research and regenerative medicine. Hypertension.

[12] Ferreira L.S., Gerecht S., Shieh H.F., Watson N., Rupnick M.A., Dallabrida S.M., Vunjak-Novakovic G., Langer R. (2007). Vascular progenitor cells isolated from human embryonic stem cells give rise to endothelial and smooth muscle like cells and form vascular networks *in vivo*. Circ. Res..

[13] Abilez O., Benharash P., Mehrotra M., Miyamoto E., Gale A., Picquet J., Xu C., Zarins C. (2006). A novel culture system shows that stem cells can be grown in 3D and under physiologic pulsatile conditions for tissue engineering of vascular grafts. J. Surg. Res..

[14] Xiong Q., Hill K.L., Li Q., Suntharalingam P., Mansoor A., Wang X., Jameel M.N., Zhang P., Swingen C., Kaufman D.S. (2011). A fibrin patch-based enhanced delivery of human embryonic stem cell-derived vascular cell transplantation in a porcine model of postinfarction left ventricular remodeling. Stem Cells.

[15] Shen G., Tsung H.C., Wu C.F., Liu X.Y., Wang X.Y., Liu W., Cui L., Cao Y.L. (2003). Tissue engineering of blood vessels with endothelial cells differentiated from mouse embryonic stem cells. Cell Res..

[16] McDevitt T.C., Ramachandran N.R. (2006). A Cellularized Biomaterial from Embryonic Stem Cells. EU Patent.

[17] Yeryemyeyev A., Artyem V., Svyetlakov A.V., Bol’shakov I.N., Shyeina J.I., Polstjanoj A.M. (2011). Method for Preparation of a Cardiomyocyte Matrix. EU Patent.

[18] Hayashi S. (2004). Blood Vessel-Specific Organogenesis from Embryonic Stem Cells on Three-Dimensional Matrigel Layer. U.S. Patent.

[19] Wernig M., Meissner A., Foreman R., Brambrink T., Ku M., Hochedlinger K. (2007). *In vitro* reprogramming of fibroblasts into a pluripotent ES-cell-like state. Nature.

[20] Yu J., Vodyanik M., Smuga-Otto K., Antosiewicz-Bourget J., Frane J.L., Tian S. (2007). Induced pluripotent stem cell lines derived from human somatic cells. Science.

[21] Stadtfeld M., Nagaya M., Utikal J., Weir G., Hochedlinger K. (2008). Induced pluripotent stem cells generated without viral integration. Science.

[22] Kim J.B., Zaehres H., Wu G., Gentile L., Ko K., Sebastiano V. (2008). Pluripotent stem cells induced from adult neural stem cells by reprogramming with two factors. Nature.

[23] Kim J.B., Zaehres H., Schöler R. (2009). Generation of Induced Pluripotent Stem (IPs) Cells. WO Patent.

[24] Yamanaka S., Tanabe K. (2009). Method for Producing Induced Pluripotent Stem Cells. WO Patent.

[25] Choi K.D., Yu J., Smuga-Otto K., Salvagiotto G., Rehrauer W., Vodyanik M., Thomson J. (2009). Hematopoietic and endothelial differentiation of human induced pluripotent stem cells. Stem Cells.

[26] Lian Q., Zhang Y., Zhang J., Zhang H.K., Wu X., Zhang Y. (2010). Functional mesenchymal stem cells derived from human induced pluripotent stem cells attenuate limb ischemia in mice. Circulation.

[27] Hibino N., Duncan D.R., Nalbandian A., Yi T., Qyang Y., Shinoka T., Breuer C.K. (2012). Evaluation of the use of an induced pluripotent stem cell sheet for the construction of tissue-engineered vascular grafts. J. Thorac. Cardiovasc. Surg..

[28] Tobiasch E., Zacharias C., Ter Horst K.W., Witt K.U. (2008). Adult Human Mesenchymal Stem Cells as Source for Future Tissue Engineering. Forschungsspitzen und Spitzenforschung.

[29] Haddouti E.M., Skroch M., Zippel N., Müller C., Birova B., Pansky A., Kleinfeld C., Winter M., Tobiasch E. (2009). Human dental follicle precursor cells of wisdom teeth: Isolation and differentiation towards osteoblasts for implants with and without scaffolds. Mater. Sci. Eng. Technol..

[30] Tobiasch E., Artmann G.M., Hescheler J., Minger S. (2010). Differentiation Potential of Adult Human Mesenchymal Stem Cells. Stem Cell Engineering.

[31] Zippel N., Schulze M., Tobiasch E. (2010). Biomaterials and mesenchymal stem cells for regenerative medicine. Recent Pat. Biotechnol..

[32] Khan D., Kleinfeld C., Winter M., Tobiasch E., Davies J. (2012). Oral Tissues as Source for Bone Regeneration in Dental Implantology. Tissue Regeneration—From Basic Biology to Clinical Application.

[33] Zhang Y., Khan D., Delling J., Tobiasch E. (2012). Mechanisms underlying the osteo- and adipo-differentiation of human mesenchymal stem cells. Sci. World J..

[34] Hass R., Kasper C., Böhm S., Jacobs R. (2011). Different populations and sources of human mesenchymal stem cells (MSC): A comparison of adult and neonatal tissue-derived MSC. Cell Commun. Signal..

[35] Dominici M., Blanc K.L., Mueller I., Slaper-Cortenbach I., Marini F.C., Krause D.S. (2006). Minimal criteria for defining multipotent mesenchymal stromal cells. The International Society for Cellular Therapy position statement. Cytotherapy.

[36] Zhang Y., Tobiasch E., Di Nardo P. (2011). The Role of Purinergic Receptors in Stem Cells in Their Derived Consecutive Tissues. Adult Stem Cell Standardization.

[37] Zippel N., Limbach C.A., Ratajski N., Urban C., Luparello C., Pansky A. (2012). Purinergic receptors influence the differentiation of human mesenchymal stem cells. Stem Cells Dev..

[38] Longo A., Librizzi M., Naselli F., Caradonna F., Tobiasch E., Luparello C. (2013). PTHrP in differentiating human mesenchymal stem cells: Transcript isoform expression, promoter methylation, and protein accumulation. Biochemie.

[39] Zuk P.A., Zhu M., Ashjian P., de Ugarte D.A., Huang J.I., Mizuno H. (2002). Human adipose tissue is a source of multipotent stem cells. Mol. Biol. Cell.

[40] Pansky A., Roitzheim B., Tobiasch E. (2007). Differentiation potential of adult of human mesenchymal stem cells. Clin. Lab..

[41] Birnbaum T., Hildebrandt J., Nuebling G., Sostak P., Straube A. (2011). Glioblastoma-dependent differentiation and angiogenic potential of human mesenchymal stem cells *in vitro*. J. Neurooncol..

[42] Kim J.H., Park I.S., Park Y., Jung Y., Kim S.H., Kim S.H. (2013). Therapeutic angiogenesis of three-dimensionally cultured adipose-derived stem cells in rat infarcted hearts. Cytotherapy.

[43] Duffy G.P., McFadden T.M., Byrne E.M., Gill S.L., Farrell E., O’Brien F.J. (2011). Towards *in vitro* vascularization of collagen-GAG scaffolds. Eur. Cells Mater..

[44] Song Y., Kamphuis M.M., Zhang Z., Sterk L.M., Vermes I., Poot A.A., Feijen J., Grijpma D.W. (2010). Flexible and elastic porous poly(trimethylene carbonate) structures for use in vascular tissue engineering. Acta Biomater..

[45] Tsigkou O., Pomerantseva I., Spencer J.A., Redondo P.A., Hart A.R., O’Doherty E., Lin Y., Friedrich C.C., Daheron L., Lin C.P. (2010). Engineered vascularized bone grafts. Proc. Natl. Acad. Sci. USA.

[46] Gong Z., Niklason L.E. (2008). Small-diameter human vessel wall engineered from bone marrow-derived mesenchymal stem cells (hMSCs). FASEB J..

[47] Kim B.S., Choi J.S., Kim J.D., Choi Y.C., Cho Y.W. (2012). Recellularization of decellularized human adipose tissue-derived extracellular matrix sheets with other human cell types. Cell Tissue Res..

[48] Zhao Y., Zhang S., Zhou J., Wang J., Zhen M., Liu Y., Chen J., Qi Z. (2010). The development of a tissue-engineered artery using decellularized scaffold and autologous ovine mesenchymal stem cells. Biomaterials.

[49] Dufourcq P., Descamps B., Tojais N.F., Leroux L., Oses P., Daret D., Moreau C., Lamazière J.M., Couffinhal T., Duplàa C. (2008). Secreted frizzled-related protein-1 enhances mesenchymal stem cell function in angiogenesis and contributes to neovessel maturation. Stem Cells.

[50] Altman A.M., Yan Y., Matthias N., Bai X., Rios C., Mathur A.B., Song Y.H., Alt E.U. (2009). IFATS collection: Human adipose-derived stem cells seeded on a silk fibroin-chitosan scaffold enhance wound repair in a murine soft tissue injury model. Stem Cells.

[51] Chung E., Nam S.Y., Ricles L.M., Emelianov S.Y., Suggs L.J. (2013). Evaluation of gold nanotracers to track adipose-derived stem cells in a PEGylated fibrin gel for dermal tissue engineering applications. Int. J. Nanomed..

[52] Matsumura G., Miyagawa-Tomita S., Shin’oka T., Ikada Y., Kurosawa H. (2003). First evidence that bone marrow cells contribute to the construction of tissue-engineered vascular autografts *in vivo*. Circulation.

[53] Cho S.W., Jeon O., Lim J.E., Gwak S.J., Kim S.S., Choi C.Y., Kim D.I., Kim B.S. (2006). Preliminary experience with tissue engineering of a venous vascular patch by using bone marrow-derived cells and a hybrid biodegradable polymer scaffold. J. Vasc. Surg..

[54] Lim S.H., Cho S.W., Park J.C., Jeon O., Lim J.M., Kim S.S., Kim B.S. (2008). Tissue-engineered blood vessels with endothelial nitric oxide synthase activity. J. Biomed. Mater. Res..

[55] Mendelson K., Aikawa E., Mettler B.A., Sales V., Martin D., Mayer J.E., Schoen F.J. (2007). Healing and remodeling of bioengineered pulmonary artery patches implanted in sheep. Cardiovasc. Pathol..

[56] Derval N., Barandon L., Dufourcq P., Leroux L., Lamazière J.M., Daret D., Couffinhal T., Duplàa C. (2008). Epicardial deposition of endothelial progenitor and mesenchymal stem cells in a coated muscle patch after myocardial infarction in a murine model. Eur. J. Cardiothorac. Surg..

[57] Ashara T., Murohara T., Sullivan A., Silver M., van der Zee R., Li T. (1997). Isolation of putative progenitor endo cells for angiogenesis. Science.

[58] Shi Q., Rafii S., Wu M.H., Wijelath E.S., Yu C., Ishida A. (1998). Evidence for circulating bone marrow-derived endothelial cells. Blood.

[59] Reyes M., Dudek A., Jahagirdar B., Koodie L., Marker P.H., Verfaillie C.M. (2002). Origin of endothelial progenitors in human postnatal bone marrow. J. Clin. Investig..

[60] Phuc P.V., Nqoc V.B., Lam D.H., Viet P.Q., Nqoc P.K. (2012). Isolation of three important types of stem cells from the same samples of banked umbilical cord blood. Cell Tissue Bank..

[61] Shirota T., He H., Yasui H., Matsuda T. (2003). Human endothelial progenitor cell-seeded hybrid graft: Proliferative and antithrombogenic potentials *in vitro* and fabrication processing. Tissue Eng..

[62] Westman J., Nilsson M., Ornitz D.M., Svahn C. (1995). Synthesis and fibroblast growth factor binding of oligosaccharides related to heparin and heparan sulphate. Carbohydr. Res..

[63] Amini A.R., Laurencin C.T., Nukavarapu S.P. (2012). Differential analysis of peripheral blood- and bone marrow-derived endothelial progenitor cells for enhanced vascularization in bone tissue engineering. J. Orthop. Res..

[64] Davis M.E., Motion J.P., Narmoneva D.A., Takahashi T., Hakuno D., Kamm R.D., Zhang S., Lee R.T. (2005). Injectable self-assembling peptide nanofibers create intramyocardial microenvironments for endothelial cells. Circulation.

[65] Sainz J., Al Haj Zen A., Caligiuri G., Demerens C., Urbain D., Lemitre M., Lafont A. (2006). Isolation of “side population” progenitor cells from healthy arteries of adult mice. Arterioscler. Thromb. Vasc. Biol..

[66] Sreerekha P.R., Divya P., Krishnan L.K. (2006). Adult stem cell homing and differentiation *in vitro* on composite fibrin matrix. Cell Prolif..

[67] Wu X., Rabkin-Aikawa E., Guleserian K.J., Perry T.E., Masuda Y., Sutherland F.W., Schoen F.J., Mayer J.E., Bischoff J. (2004). Tissue-engineered microvessels on three-dimensional biodegradable scaffolds using human endothelial progenitor cells. Am. J. Physiol. Heart Circ. Physiol..

[68] Allen J., Khan S., Serrano M.C., Ameer G. (2008). Characterization of porcine circulating progenitor cells: Toward a functional endothelium. Tissue Eng. Part A.

[69] Cherqui S., Kingdon K.M., Thorpe C., Kurian S.M., Salomon D.R. (2007). Lentiviral gene delivery of vMIP-II to transplanted endothelial cells and endothelial progenitors is proangiogenic *in vivo*. Mol. Ther..

[70] Schmeckpeper J., Ikeda Y., Kumar A.H., Metharom P., Russell S.J., Caplice N.M. (2009). Lentiviral tracking of vascular differentiation in bone marrow progenitor cells. Differentiation.

[71] Fioretta E.S., Fledderus J.O., Baaijens F.P., Bouten C.V. (2012). Influence of substrate stiffness on circulating progenitor cell fate. J. Biomech..

[72] Clever Y.P., Cremers B., Krauss B., Böhm M., Speck U., Laufs U., Scheller B. (2011). Paclitaxel and sirolimus differentially affect growth and motility of endothelial progenitor cells and coronary artery smooth muscle cells. EuroIntervention.

[73] Cottone R.J., Yoklavich M., Parker S. (2011). Progenitor Endothelial Cell Capturing with a Drug Eluting Implantable Medical Device. U.S. Patent.

[74] Melero-Martin J.M., Khan Z.A., Picard A., Wu X., Paruchuri S., Bischoff J. (2007). *In vivo* vasculogenic potential of human blood-derived endothelial progenitor cells. Blood.

[75] Griese D.P., Ehsan A., Melo L.G., Kong D., Zhang L., Mann M.J., Pratt R.E., Mulligan R.C., Dzau V.J. (2003). Isolation and transplantation of autologous circulating endothelial cells into denuded vessels and prosthetic grafts: Implications for cell-based vascular therapy. Circulation.

[76] He H., Shirota T., Yasui H., Matsuda T. (2003). Canine endothelial progenitor cell-lined hybrid vascular graft with nonthrombogenic potential. J. Thorac. Cardiovasc. Surg..

[77] Yu J., Wang A., Tang Z., Henry J., Li-Ping L.B., Zhu Y., Yuan F., Huang F., Li S. (2012). The effect of stromal cell-derived factor-1α/heparin coating of biodegradable vascular grafts on the recruitment of both endothelial and smooth muscle progenitor cells for accelerated regeneration. Biomaterials.

[78] Noishiki Y., Seki F., Yasuhiro A., Akihiro S.Y. (2005). Method for Harvesting Bone Marrow and Its Medical Apparatus. U.S. Patent.

[79] Muschler G.F. (2006). Apparatus and Method for Harvesting Bone Marrow. U.S. Patent.

[80] Sciorra L. (2012). Multipotent Stem Cell-Based Culture Systems and Models. WO Patent.

[81] Harman R.J., Sand T.T. (2004). Methods of Preparing and Using Stem Cell Compositions and Kits Comprising the Same. EU Patent.

[82] Kieda C., Grillon C., Lamerant-Fayel N., Paprocka M., Krawczenko A., Goszyk-Dus D. (2011). Human and Murine Stem-Cell Lines: Models of Endothelial Cell Precursors. EU Patent.

[83] Ma T., Junho K. (2011). Mesenchymal Stem Cells (MSC) Expansion Methods and Materials. WO Patent.

[84] Xianqun F., Yefei W., Huifang Z. (2010). Method for *in-Vitro* Fusion of Stem Cells and Porous Biomaterial. CN Patent.

[85] Gostjeva E.V., Thilly W.G. (2011). Wound Healing Metakaryotic Stem Cells and Methods of Use Thereof. WO Patent.

[86] Amoh Y., Li L., Yang M., Jiang P. (2004). Angiogensis Models Using Nestin-Expressing Stem Cells to Image Nascent Blood Vessels. U.S. Patent.

[87] Lee J.W., Park M.S.K., Yun H. (2011). Method for Inducing *in Vivo* Migration of Stem Cells. WO Patent.

[88] Hantash B. (2009). Differentiation of Mesenchymal Stem Cells into Fibroblasts, Compositions Comprising Mesenchymal Stem Cell-Derived Fibroblasts, and Methods of Using the Same. WO Patent.

[89] Guoqi T., Yigang W., Baisong Z. (2012). Technique for Heart Disease External Differentiation Therapy by Utilizing Stem Cells Ofmasticatory Muscles and Orbicularis Oculi Muscles. CN Patent.

[90] Nanxue S.L., Lidong G., Xuetao P., Cixian B., Fan Y., Yunfang W., Huimin Y. (2005). Structural Method and Application of Tissue Engineering Adipose Tissue. CN Patent.

[91] Shortkroff S., Khoury J., Tarrant L.J.B., Claesson H.P.I., Smith R.L. (2008). A Method for Improvement of Differentiation of Mesenchymal Stem Cells Using a Double-Structured Tissue Implant. EU Patent.

[92] Park K., Deok K., Hee J.H., Dong K., Hong Y., Jin C., Heung J., Jang J.W. (2009). Method for Surface Modification of Polymeric Scaffold for Stem Cell Transplantation Using Decellularized Extracellular Matrix. U.S. Patent.

[93] Aicher W., Angres B. (2010). Isolierung von Mesenchymalen Stammzellen. DE Patent.

[94] Hamada Y., Matsumura N., Egusa H., Kaneda Y., Okazaki M. (2007). Mesenchymal Cell Proliferation Stimulator and Skeletal System Biomaterial. WO Patent.

[95] Cho M. (2011). Scaffold for Articular Cartilage Regeneration and Method for Manufacturing Same. U.S. Patent.

[96] Komeno K., Ono S., Tanne K. (2004). Material for Graft and Method for Culturing Anaplastic Mesenchymal Stem Cell. JP Patent.

[97] Huang L.H. (2010). Cell Tissue Gel Containing Collagen and Hyaluronan. EU Patent.

[98] Abatangelo G., Callegaro L. (1996). A Biologic Material Comprising an Efficient Culture of Bone Marrow Stem Cells Partially or Completely Differentiated into Connective Tissue Cells and a Three-Dimensional Biocompatible and Biodegradable Matrix Consisting of a Hyaluronic Acid Derivative. EU Patent.

[99] Choi Y.S., Noh S., Eun L., Youngjun J., Sang Y., Kim S.M., Han K., Chung H.M. (2012). Cartilage Cell Treating Agent Comprising Collagen, Hyaluronic Acid Derivative, and Stem Cell Derived from Mammal Umbilical Cord. WO Patent.

[100] Min B., Hyoun P., So R., Park S.H. (2007). Method for Differenciating Mesenchymal Stem Cell and Culturing Chondrocytes Using Alginate Coated Fibrin/Ha Composite Scaffold. KR Patent.

[101] Shimizu T., Haraguchi Y., Aoki S., Okano M., Ono T., Rin K., Horii A. (2011). Differentiation Method to Smooth Muscle Cell of Mesenchymal Stem Cell, Production Method of Smooth Muscle Implant, and Smooth Muscle Implant. JP Patent.

[102] Westenfelder C. (2012). Therapeutic Method for Kidney Disease and Multi Organ Failure due to Mesenchymal Stem Cell, and Mesenchymal Stem Cell-Conditioned Medium. JP Patent.

[103] Arai K. (2006). Method for Producing Collagen, Cell and DNA. JP Patent.

[104] Brouard S., Otterbein L.E., Anrather J., Tobiasch E., Bach F.H., Choi A.M., Soares M.P. (2000). Carbon monoxide generated by hemeoxygenase 1 suppresses endothelial cell apoptosis. J. Exp. Med..

[105] Brouard S., Berberat P.O., Tobiasch E., Seldon M.P., Bach F.H., Soares M.P. (2002). Hemeoxygenase-1-derived carbon monoxide requires the activation of transcription factor NF-kappa B to protect endothelial cells from tumor necrosis factor-alpha-mediated apoptosis. J. Biol. Chem..

[106] Soares M.P., Usheva A., Brouard S., Berberat P.O., Gunther L., Tobiasch E., Bach F.H. (2002). Modulation of endothelial cell apoptosis by heme oxygenase-1-derived carbon monoxide. Antioxid. Redox Signal..

[107] Jaffe E.A., Nachman R.L., Becker C.G., Minich C.R. (1973). Culture of human endothelial cells derived from umbilical veins. Identification by morphologic and immunologic criteria. J. Clin. Investig..

[108] Voyta J.C., Via D.P., Butterfield C.E., Zetter B.R. (1984). Identification and isolation of endothelial cells based on their increased uptake of acetylated-low density protein. J. Cell Biol..

[109] Knighton D.R. (1990). Method for Isolating Wound Capillary Endothelial Cells. WO Patent.

[110] Aldons J.L. (2000). Atherosclerosis. Nature.

[111] Ray J.L., Leach R., Herbert J.M., Bentson M. (2001). Isolation of vascular smooth muscle cells from a single murine aorta. Methods Cell Sci..

[112] Ribeiro M.P., Relvas R., Chiquita I.J. (2010). Isoalation of human umbilical artery smooth muscle cells (HUASMC). J. Vis. Exp..

[113] Fillinger M.F., O’Connor S.E., Wagner R.J., Cronenwett J.L. (1993). The effect of endothelial cell coculture on smooth muscle cell proliferation. J. Vasc. Surg..

[114] Wallace C.S., Truskey G.A. (2010). Direct-contact co-culture between smooth muscle cells and endothelial cells inhibits TNF-alpha-mediated endothelial cell activation. Am. J. Physiol. Heart Circ. Physiol..

[115] Chen L., Xi T., Yang Z., Wang W., Liu Q., Wu Y., Gu Y., Wang J., Feng Z. (2005). Effect of surface property of different polyether-ester copolymers on growth of smooth muscle cells and endothelial cells. Zhongguo Xiu Fu Chong Jian Wai Ke Za Zhi.

[116] Baguneid M., de Mel A., Yildirimer L., Fuller B.J., Hamilton G., Seifalian A.M. (2011). *In vivo* study of a model tissue-engineered small-diameter vascular bypass graft. Biotechnol. Appl. Biochem..

[117] Boni L., Chalajour F., Sasaki T., Snyder R.L., Boyd W.D., Riemer R.K., Reddy V.M. (2012). Reconstruction of pulmonary artery with porcine small intestinal submucosa in a lamb surgical model: Viability and growth potential. J. Thorac. Cardiovasc. Surg..

[118] Callegari A., Bollini S., Iop L., Chiavegato A., Torregrossa G., Pozzobon M., Gerosa G., de Coppi P., Elvassore N., Sartore S. (2007). Neovascularization induced by porous collagen scaffold implanted on intact and cryoinjured rat hearts. Biomaterials.

[119] Saif J., Schwarz T.M., Chau D.Y., Henstock J., Sami P., Leicht S.F., Hermann P.C., Alcala S., Mulero F., Shakesheff K.M. (2010). Combination of injectable multiple growth factor-releasing scaffolds and cell therapy as an advanced modality to enhance tissue neovascularization. Arterioscler. Thromb. Vasc. Biol..

[120] Stoeckius M., Erat A., Fujikawa T., Hiromura M., Koulova A., Otterbein L., Bianchi C., Tobiasch E., Dagon Y., Sellke F.W. (2012). Essential roles of Raf/ERK/MAPK pathway, YY1, and Ca^+2^ influx in the growth arrest of human vascular smooth muscle cells by bilirubin. J. Biol. Chem..

[121] Nabel E.G., Nabel G.J. (1995). Inhibition of Arterial Smooth Muscle Cell Proliferation. EU Patent.

[122] Suda T., Katagiri T., Kodaira K., Goto M., Higashio K. (2006). Therapeutic Agent for Arteriosclerosis. WO Patent.

[123] Wolinsky H. (1986). Method for the Prevention of Restenosis. U.S. Patent.

[124] Altman P. (2011). Method of Treating Coronary Arteries with Perivascular Delivery of Therapeutic Agents. U.S. Patent.

[125] Edelman E., Nathan A., Nugent M.A. (1996). Inhibition of Vascular Occlusion Following Vascular Intervention. EU Patent.

[126] Uchida S. (2012). Vascular Prothesis. WO Patent.

[127] Takano T. (1991). Artificial Blood Vessel. JP Patent.

[128] Cao Y.L. (2002). Method and Apparatus for Constructing Blood Vessel *in Vitro* in the Tissue Project. CN Patent.

[129] Burleigh D. (1994). Therapeutic Treatment for Inhibiting Blood Vessel Blockage Using a Polypeptide. EP Patent.

[130] Jianhong M., Chunli H., Chuhong Z., Dajun Y., Li L. (2007). Biological Artificial Blood Vessel Capable of *in Vivo* Capturing Endothelial Ancestral Cell. CN Patent.

[131] Iwazawa R., Nakamura K. (2012). Scaffold for Vascular Endothelial Cell Migration. EU Patent.

[132] Hoganson D.M., Vacanti J.P. (2009). System and Method for *in Vitro* Blood Vessel Modeling. WO Patent.

[133] Kellar R.S., Landeen L.K., Shepherd B.R., Naughton G.K., Ratcliffe A., Williams S.K. (2001). Scaffold-based three-dimensional human fibroblast culture provides a structural matrix that supports angiogenesis in infarcted heart tissue. Circulation.

[134] Tosun Z., McFetridge P.S. (2005). Improved recellularization of *ex vivo* vascular scaffolds using direct transport gradients to modulate ECM remodeling. FASEB J..

[135] Formigli L., Perna A.M., Meacci E., Cinci L., Margheri M., Nistri S., Tani A., Silvertown J., Orlandini G., Porciani C. (2007). Paracrine effects of transplanted myoblasts and relaxin on post-infarction heart remodelling. J. Cell. Mol. Med..

[136] Pařízek M., Novotná K., Bačáková L. (2011). The role of smooth muscle cells in vessel wall pathophysiology and re-construction using bioactive synthetic polymers. Physiol. Res..

[137] Chlupač J., Filova E., Bacakova L. (2009). Blood vessel replacement: 50 Years of development and tissue engineering paradigms in vascular surgery. Physiol. Res..

[138] Engler A., Sen S., Sweeney H., Discher D. (2006). Matrix elasticity directs stem cell lineage specification. Cell.

[139] Gilbert P., Havenstritte K., Magnusson K., Sacco A., Leonardi N., Kraft P., Nguyen N., Thrun S., Lutolf M., Blau H. (2010). Substrate elasticity regulates skeletal muscle stem cell self-renewal in culture. Science.

[140] Holst J., Watson S., Lord M.S., Eamegdool S.S., Bax D.V., Nivison-Smith L.B., Kondyurin A., Ma L., Oberhauser A.F., Weiss A.S. (2010). Substrate elasticity provides mechanical signals for the expansion of hemopoietic stem and progenitor cells. Nat. Biotechnol..

[141] Benoit D., Schwartz M., Durney A., Anseth K. (2008). Small functional groups for controlled differentiation of hydrogel-encapsulated human mesenchymal stem cells. Nat. Mater..

[142] Schmedlen R., Elbjeirami W., Gobin A., West J. (2003). Tissue engineered small-diameter vascular grafts. Clin. Plast. Surg..

[143] Sawhney A., Pathak C., Hubbel J. (1993). Bioerodible hydrogels based on photopolymerized poly(ethylene glycol)-*co*-poly(α-hydroxy acid)diacrylate macromers. Macromelecules.

[144] Szycher M. (2013). Szycher’s Handbook of Polyurethane.

[145] Wright J. Using Polyurethanes in Medical Applications. http://www.mddionline.com/article/using-polyurethanes-medical-applications.

[146] Baer G., Wilson T., Matthews D., Maitland D. (2007). Shape-memory behavior of thermally stimulated polyurethane for medical applications. J. Appl. Polym. Sci..

[147] McBane J.E.M., Sharifpoor S., Labow R.S., Ruel M., Suuronen E.J., Santerre J.P. (2012). Tissue engineering a small diameter vessel substitute: Engineering constructs with select biomaterials and cells. Curr. Vasc. Pharmacol..

[148] Romaškevič T., Budrienė S., Pielichowski K., Pielichowski J. (2006). Application of polyurethane-based materials for immobilization of enzymes and cells: A review. Chemija.

[149] Prewitz M., Seib F., Pompe T., Werner C. (2012). Polymeric biomaterials for stem cell bioengineering. Macromol. Rapid Commun..

[150] Szilagyi D., France L., Smith R. (1958). Clinical use of an elastic Dacron prosthesis. Arch. Surg..

[151] Van Damme H., Deprez M., Creemers E., Limet R. (2005). Intrinsic structural failure of polyester (dacron) vascular grafts. A general review. Acta Chir. Belg..

[152] Takamoto T., Ichinohe N., Tabata Y. (2012). Proliferation of rat mesenchymal stem cells in collagen sponges reinforced with poly(ethylene terephthalate) fibers by stirring culture method. J. Biomater. Sci. Polym..

[153] Prokoph S., Chavakis E., Levental K., Zieris A., Freudenberg U., Dimmeler S., Werner C. (2012). Sustained delivery of SDF-1α from heparin-based hydrogels to attract circulating pro-angiogenic cells. Biomaterials.

[154] Kurane A., Vyavahare N. (2009). *In vivo* vascular tissue engineering: Influence of cytokine and implant location on tissue specific cellular recruitment. J. Tissue Eng. Regen. Med..

[155] Huang N.F., Li S. (2011). Regulation of the matrix microenvironment for stem cell engineering and regenerative medicine. Ann. Biomed. Eng..

[156] Prewitz M.K., Seib P.F., von Bonin M., Friedrichs J., Stibel A., Niehage C., Müller K., Anastassiadis K., Waskow C., Hoflack B. (2013). Tightly anchored tissue-mimetic matrices as instructive stem cell microenvironments. Nat. Methods.

[157] Embuscado M.E., Huber K.C. (2009). Edible Films and Coatings for Food Applications.

[158] Le Ricousse-Roussanne S., Barateau V., Contreres J.O., Boval B., Kraus-Berthier L., Tobelem G. (2004). *Ex vivo* differentiated endothelial and smooth muscle cells from human cord blood progenitors home to the angiogenic tumor vasculature. Cardiovasc. Res..

[159] Bohrer C., Ruth P., Schatton W. (2005). Verfahren zur Herstellung von Porösen Schwämmen aus Gereinigtem Marinen Kollagen. DE Patent.

[160] Donzelli E., Salvade A., Mimo P., Vigano M., Morrone M., Papagna R., Carini F., Zaopo A., Miloso M., Baldoni M. (2007). Mesenchymal stem cells cultured on a collagen scaffold: *In vitro* osteogenic differentiation. Arch. Oral Biol..

[161] Ruszczak Z. (2003). Effect of collagen matrices on dermal wound healing. Adv. Drug Deliv. Rev..

[162] Chvapil M. (1977). Collagen sponge: Theory and practice of medical applications. J. Biomed. Mater. Res..

[163] Casu B., Lindahl U. (2001). Structure and biological interactions of heparin and heparan sulfate. Adv. Carbohydr. Chem. Biochem..

[164] Avci F.Y., de Angelis P.L., Liu J., Linhardt R.J., Demchenko A.V. (2007). Enzymatic Synthesis of Glycosaminoglycanes: Improving on Nature, Frontiers in Modern Carbohydrate Chemistry. ACS Symposium Series 960.

[165] Linhardt R.J., Claude S. (2003). Hudson award address in carbohydrate chemistry. Heparin: Structure and activity. J. Med. Chem..

[166] Jaques L.B. (1979). Heparin: An old drug with a new paradigm. Science.

[167] Linhardt R.J. (1991). Heparin: An important drug enters its seventh decade. Chem. Ind..

[168] Dougher A.M., Wasserstrom H., Torley L., Shridaran L., Westdock P., Hileman R.E., Fromm J.R., Anderberg R., Lyman S., Linhardt R.J. (1997). Identification of a heparin binding peptide on the extracellular domain of the KDR VEGF receptor. Growth Factors.

[169] Lucas H., Basten J.E., van Dinther T.G., Meuleman D.G., van Aelst S.F., van Boeckel C.A. (1990). Syntheses of heparin-like pentamers containing opened uronic acid moieties. Tetrahedron.

[170] Van Boeckel C.A., Petitou M. (1993). The unique antithrombin III binding domain of heparin: A lead to new synthetic antithrombotics. Angew. Chem. Int. Ed..

[171] Karst N.A., Linhardt R.J. (2003). Recent chemical and enzymatic approaches to the synthesis of glycosaminoglycan oligosaccharides. Curr. Med. Chem..

[172] Bacakova L., Svorcik V., Kimura D. (2008). Cell Colonization Control by Physical and Chemical Modification of Materials. Cell Growth Processes: New Research.

[173] Sachlos E., Czernuszka J. (2003). Making tissue engineering scaffolds work. Eur. Cells Mater..

[174] Moroni L., Wijn J., van Blitterswijkc A. (2008). Integrating novel technologies to fabricate smart scaffolds. J. Biomater. Sci. Polym. Ed..

[175] Anderson S.B., Lin C.C., Kuntzler D.V., Anseth K.S. (2011). The performance of human mesenchymal stem cells encapsulated in cell-degradable polymer-peptide hydrogels. Biomaterials.

[176] Wan A.C.A., Ying J.Y. (2010). Nanomaterials for *in situ* cell delivery and tissue regeneration. Adv. Drug Deliv. Rev..

[177] Ma P.X. (2008). Biomimetic materials for tissue engineering. Adv. Drug Deliv. Rev..

[178] Goldstein A., Zhu G., Morris G. (1999). Effect of osteoblastic culture conditions on the structure of poly(d,l-lactic-*co*-glycolic acid) foam scaffolds. Tissue Eng..

[179] Nasibulin A.G., Anisimov A.S., Pikhitsa P.V., Jiang H., Brown D.P., Choi M., Kauppinen E.I. (2007). Investigations of nanobud formation. Chem. Phys. Lett..

[180] Bauer S., Park J., von der Mark K., Schmuki P. (2008). Improved attachment of mesenchymal stem cells on super hydrophobic TiO_2_ nanotubes. Acta Biomater..

[181] Park J., Bauer S., Schmuki P. (2009). Narrow window in nanoscale dependent activation of endothelial cell growth and differentiation on TiO_2_ nanotube surfaces. Nano Lett..

[182] Zhang L., Webster T. (2009). Nanotechnology and nanomaterials: Promises for improved tissue regeneration. Nano Today.

[183] Kim K., Dean D., Lu A.Q. (2011). Early osteogenic signal expression of rat bone marrow stromal cells is influenced by both hydroxyapatite nanoparticles content and initial cell seeding density in biodegradable nanocomposites scaffolds. Acta Biomater..

[184] Oliveira J.M., Sousa R.A., Malafaya P.B. (2011). *In vivo* study of dendron-like nanoparticles for stem cells “tune-up”: From nano to tissues. Nanomedicine.

[185] Wang J., Yu X. (2010). Preparation, characterization and *in vitro* analysis of novel structured nanofibrous scaffolds for bone tissue engineering. Acta Biomater..

[186] Kretlow J., Mikos A. (2008). From material to tissue: Biomaterial development, scaffold fabrication, and tissue engineering. AIChE J..

[187] Wei G., Ma P.X. (2008). Nanostructured biomaterials for regeneration, nano-scaled drug release systems incorporated into nanostructured biomaterials represents a novel and promising strategy to tissue regeneration. Adv. Funct. Mater..

[188] Burdick J.A., Vunjak-Novakovic G. (2009). Engineered microenvironments for controlled stem cell differentiation. Tissue Eng..

[189] Peltola S., Sanna M., Melchels F., Grijpma D., Kellomaki M. (2008). A review of rapid prototyping techniques for tissue engineering purposes. Ann. Med..

[190] Bens A., Seitz H., Bermes H., Emons M., Pansky A., Roitzheim B. (2007). Non-toxic flexible photopolymers for medical stereolithography technology. Rapid Prototyp. J..

[191] Sitharaman B., Avti P.K., Schaefer K. (2011). A novel nanoparticle-enhanced photoacustic stimulus for bone tissue engineering. Tissue Eng. Part A.

[192] Lim Y.C., Johnson J., Fei Z.Z. (2011). Micropatterning and characterization of electrospun poly(epsilon-caprolactone)/gelatin nanofiber tissue scaffolds by femtosecond laser ablation for tissue engineering applications. Biotechnol. Bioeng..

[193] Fan D.M., Akkaraju G.R., Couch E.F. (2011). The role of nanostructured mesoporous silicon in discriminating *in vitro* calcification for electrospun composite tissue engineering scaffolds. Nanoscale.

[194] Qiang L., Qing L.F. (2006). Preparation of 3-D regenerated fibroin scaffolds with freeze drying method and freeze drying/foaming technique. J. Mater. Sci. Mater. Med..

[195] Gagnieu C. (1998). Vernetzbare Kollagenderivate, Verfahren zu Ihrer Herstellung und Ihre Verwendung zur Herstellung von Biomaterialien. DE Patent.

[196] Zeugolis D.I., Paul G.R., Attenburrow G. (2009). Cross-linking of extruded collagen fibers: A biomimetic three-dimensional scaffold for tissue engineering applications. J. Biomed. Mater. Res. Part A.

[197] Fratzl P. (2008). Collagen—Structure and Mechanics.

[198] Kling S. (2009). Biomechanical Response of Normal and Cross-Linked Corneas.

[199] Nimni M.E., Wise L., Trantolo D.J., Altobelli D.E., Yaszemski M.J. (1995). Collagen: Molecular Structure and Biomaterial Properties. Encyclopedic Handbook of Biomaterials and Bioengineering: Part A: Materials.

[200] Khor E. (1997). Methods for the treatment of collagenous tissues for bioprostheses. Biomaterials.

[201] Pischinger A., Karl F. (2010). Das System der Grundregulation—Grundlagen Einer Ganzheitsbiologischen Medizin.

[202] Reich G. (1966). Kollagen: Eine Einführung in Methoden, Ergebnisse und Probleme der Kollagenforschung.

[203] Hamaguchi P.Y., Shiku Y., Tanaka M. (2003). Property improvement of fish water soluble protein films by dialdehyde starch (DAS) and/or sodium dodecyl sulfate (SDS) treatments. J. Package Technol. Sci..

[204] Audic J.-L., Chaufer B. (2010). Caseinate based biodegradable films with improved water resistance. J. Appl. Polym. Sci..

[205] Angele P., Abke J., Kujat R., Faltermeier H., Schumann D., Nerlich M., Kinner B., Englert C., Ruszczak Z., Mehrl R. (2004). Influence of different collagen species on physico-chemical properties of cross-linked collagen matrices. Biomaterials.

[206] Sommer I., Kunz P.M. (2012). Improving the water resistance of biodegradable collagen films. J. Appl. Polym. Sci..

[207] Renner M.U. (2004). Gelatinefilme als Arzneistoffträger.

[208] Gratzer P.F., Pereira C.A., Lee J.M. (1996). Solvent environment modulates effects of glutaraldehyde crosslinking on tissue-derived biomaterials. J. Biomed. Mater. Res..

[209] Jayakrishnan A., Jameela S.R. (1996). Glutaraldehyde as a fixative in bioprostheses and drug delivery matrices. Biomaterials.

[210] Burness D.M., Pouradier J., James T.H. (1977). The Hardening of Gelatin and Emulsions. The Theory of Photographic Process.

[211] Petite H., Rault I., Huc A., Menasche P., Herbage D. (1990). Use of the acyl azide method for cross-linking collagen-rich tissues such as pericardium. J. Biomed. Mater. Res..

[212] Marquié C. (2001). Chemical reactions in cottonseed protein cross-linking by formaldehyde, glutaraldehyde, and glyoxal for the formation of protein films with enhanced mechanical properties. J. Agric. Food Chem..

[213] Cheung D.T., Nimni M.E. (1982). Mechanism of crosslinking of proteins by glutaraldehyde II. Reaction with monomeric and polymeric collagen. Connect. Tissue Res..

[214] Damink L.H., Dijkstra P.J., van Luyn M.J.A., van Wachem P.B., Nieuwenhuis P., Feijen J. (1996). Crosslinking of dermal sheep collagen using a water-soluble carbodiimide. Biomaterials.

[215] Rault I., Frei V., Herbage D. (1996). Evaluation of different chemical methods for cross-linking collagen gel, films and sponges. J. Mater. Sci. Mater. Med..

[216] Bedino J.H. (2003). Champion—An Expanding Encyclopedia of Mortuary Practices.

[217] Maser F. (1996). Wursthüllen auf Basis von Kollagen. 1. Freiberger Kollagensymposium.

[218] Marzec E., Pietrucha K. (2008). The effect of different methods of cross-linking of collagen on its dielectric properties. Biophys. Chem..

[219] Stachel I., Schwarzenbolz U., Henle T., Meyer M. (2010). Cross-linking of type I collagen with microbial transglutaminase: Identification of cross-linking sites. Biomacromol.

[220] Chambi H., Grosso C. (2006). Edible films produced with gelatin and casein crosslinked with transglutaminase. Food Res. Int..

[221] Taylor M.M., Marmer W.N., Brown E.M. (2005). Characterization of biopolymers prepared from gelatin and sodium caseinate for potential use in leather processing. J. Am. Leather Chem. Assoc..

[222] Marx C. (2008). Optimierung Einer Mikrobiellen Transglutaminase Mittels Random Mutagenese. Dissertation.

[223] Kaminski A., Grazka E., Jastrzebska A., Marowska J., Gut G., Wojciechowski A. (2012). Effect of accelerated electron beam on mechanical properties of human cortical bone: Influence of different processing methods. Cell Tissue Bank..

[224] Weadock K., Olson R.M., Silver F.H. (1983). Evaluation of collagen crosslinking techniques. Biomater. Med. Devices Artif. Organs.

[225] Kamińska A., Sionkowska A. (1996). Effect of UV radiation on the infrared spectra of collagen. Polym. Degrad. Stab..

[226] Raiskup F., Spoerl E. (2013). Corneal crosslinking with riboflavin and ultraviolet AI principles. Ocul. Surf..

[227] Hayes S., Kamma-Lorger C.S., Boote C., Young R.D., Quantock A.J. (2013). The effect of riboflavin/UVA collagen cross-linking therapy on the structure and hydrodynamic behaviour of the ungulate and rabbit corneal stroma. PLoS One.

[228] Cheung D.T., Perelman N., Tong D., Nimni M.E. (2004). The effect of γ-irradiation on collagen molecules, isolated α-chains, and crosslinked native fibers. J. Biomed. Mater. Res..

[229] Drobny J.G. (2010). Radiation Technology for Polymers.

[230] Cheng S. (2011). Radiation Processing of Polymer Materials and Its Industrial Applications.

[231] Jiang B., Wu Z., Zhao H., Tang F., Lu J., Wie Q., Zhang X. (2006). Electron beam irradiation modification of collagen membranes. Biomaterials.

[232] Seto A., Gatt C.J., Dunn M.G. (2008). Radioprotection of tendon tissue via crosslinking and free radical scavenging. Clin. Orthop. Relat. Res..

[233] Zuwei M., Zhengwei M., Gao C. (2007). Surface modification and property analysis of biomedical polymers used for tissue engineering. Colloid Surf. B.

[234] Charlesby A. (1977). Use of high energy radiation for crosslinking and degradation. Radiat. Phys. Chem..

[235] Menges G. (1998). Werkstoffkunde Kunststoffe.

[236] Flory P.J., Rehner J. (1943). Statistical mechanics of cross-linked polymer networks II. Swelling. J. Chem. Phys..

[237] Gottlieb R., Kaiser C., Gohs U., Arndt K.F. (2007). Temperature sensitive hydrogels based on hydroxypropyl cellulose by high energy irradiation. Macromol. Symp..

[238] Lappan U., Geibler U., Gohs U., Uhlmann S. (2009). Influence of irradiation temperature on grafting of styrene into poly(tetrafluoroethylene-*co*-hexafluoropropylene) films. Macromol. Mater. Eng..

[239] Abke J. (2003). Verbesserung der Biokompatibilität Metallischer Implantate Durch Kovalente Anbindung Einer Quervernetzten Kollagenschicht. Dissertation.

[240] Dahl B.J., Spotts E., Truong J.Q. (2012). Corneal collagen crosslinking: An introduction and literature review. Optometry.

[241] Suljovrujic E., Ignjatovic N., Uskokovic D. (2003). Gamma irradiation processing of hydroxylapatite/poly-l-lactide composite biomaterial. Radiat. Phys. Chem..

[242] Heger A., Dorschner H., Dunsch L., Ihme B., Lunkwitz K. (1990). Technologie der Strahlenchemie von Polymeren.

[243] Hirose M., Yamato M., Kwon O.H., Harimoto M., Kushida A., Shimizu T., Kikushi A., Okano T. (2000). Temperature-responsive surface for novel co-culture systems of hepatocytes with endothelial celle: 2-D pattern and double layered co-cultures. Yonsei Med. J..

[244] Röthemeyer F., Sommer F. (2006). Kautschuktechnologie.

[245] Becker G.W., Braun D. (1990). Kunststoffhandbuch.

[246] Khorasani M.T., Mirzadeh H., Irani S. (2007). Plasma surface modification of poly(l-lactic acid) and poly(lactic-*co*-glycolic acid) films for improvement of nerve cells adhesion. Radiat. Phys. Chem..

[247] Leonard D., Pick L.T., Farrar D.F., Dickson G.R., Orr J.F., Buchanan F.J. (2009). The modification of PLA and PLGA using electron-beam radiation. J. Biomed. Mater. Res..

[248] Loo S.C.J., Ooi C.P., Boey Y.C.F. (2006). Influence of electron-beam radiation on the hydrolytic degradation behavior of poly(lactide-*co*-glycolide) (PGLA). Biomaterials.

[249] Miao P., Wu D., Zhao C., Xu G., Zeng K., Wang Y., Fu Q., Yang G. (2010). Modification of poly(d,l-lactic acid)-*co*-poly(ethylene glycol) copolymer by low energy electron beam (EB) radiation. e-Polymers.

[250] Quynh T.M., Mitomo H., Nagasawa N., Wada Y., Yoshii F., Tamada M. (2007). Properties of crosslinked polylactides (PLLA & PDLA) by radiation and its biodegradability. Eur. Polym. J..

[251] Rasal R.M., Janorkar A.J., Hirt D.E. (2010). Poly(lactic acid) modifications. Prog. Polym. Sci..

[252] Wang S., Cui W., Bei J. (2005). Bulk and surface modifications of polylactide. Anal. Bioanal. Chem..

[253] Heinrich G., Straube E., Helmis G. (1988). Rubber elasticity of polymer networks: Theories. Adv. Polym. Sci..

[254] Neuhaus-Steinmetz H. Penetration Depth of the Radiation Dose and Dose Yield for Low Energy Electron Beam Accelerators. Proceedings of the Radtech Europe.

[255] Sterilisation von Produkten für die Gesundheitsfürsorge—Strahlen Teil 2: Festlegung der Sterilisationsdosis. http://www.rki.de/DE/Content/Infekt/Krankenhaushygiene/Kommission/Downloads/Medprod_Rili_2012.pdf?__blob=publicationFile.

[256] Heilmann A., Schwegmann C.H. (2010). Beschleunigte elektronen sterilisieren materialschonend. Dev. Med..

[257] Cleland M.R. (1983). Radiation processing: Basic concepts and practical aspects. J. Ind. Radiat. Technol..

[258] Hiraoka Y., Kimura Y., Ueda H., Tabata Y. (2003). Fabrication and biocompatibility of collagen sponge reinforced with poly(glycolic acid) fiber. Tissue Eng..

[259] Melchiorri A.J., Hibino N., Fisher J.P. (2013). Strategies and techniques to enhance the *in situ* endothelialization of small-diameter biodegradable polymeric vascular grafts. Tissue Eng. Part B.

[260] Perán M., García M.A., Lopez-Ruiz E., Jiménez G., Marchal J.A. (2013). How can nanotechnology help to repair the body? Advances in cardiac, skin, bone, cartilage, nerve and cardiac tissue regeneration. Materials.

